# Unified theory of Alzheimer’s disease (UTAD): implications for prevention and curative therapy

**DOI:** 10.1186/s40303-016-0018-8

**Published:** 2016-07-15

**Authors:** Michael Nehls

**Affiliations:** Independent Researcher, Allmendweg 1, 79279 Vörstetten, Germany

**Keywords:** Unified theory Alzheimer’s disease (UTAD), Grandmother-hypothesis (GMH), Neuronal rejuvenation (NRJ), Adult hippocampal neurogenesis (AHN), Law of the minimum (LOM), Curative AD therapy, Causal AD prevention

## Abstract

The aim of this review is to propose a Unified Theory of Alzheimer’s disease (UTAD) that integrates all key behavioural, genetic and environmental risk factors in a causal chain of etiological and pathogenetic events. It is based on three concepts that emanate from human’s evolutionary history: (1) The grandmother-hypothesis (GMH), which explains human longevity due to an evolutionary advantage in reproduction by trans-generational transfer of acquired knowledge. Consequently it is argued that mental health at old-age must be the default pathway of humans’ genetic program and not development of AD. (2) Therefore, mechanism like neuronal rejuvenation (NRJ) and adult hippocampal neurogenesis (AHN) that still function efficiently even at old age provide the required lifelong ability to memorize personal experiences important for survival. Cumulative evidence from a multitude of experimental and epidemiological studies indicate that behavioural and environmental risk factors, which impair productive AHN, result in reduced episodic memory performance and in reduced psychological resilience. This leads to avoidance of novelty, dysregulation of the hypothalamic–pituitary–adrenal (HPA)-axis and cortisol hypersecretion, which drives key pathogenic mechanisms of AD like the accumulation and oligomerization of synaptotoxic amyloid beta, chronic neuroinflammation and neuronal insulin resistance. (3) By applying to AHN the law of the minimum (LOM), which defines the basic requirements of biological growth processes, the UTAD explains why and how different lifestyle deficiencies initiate the AD process by impairing AHN and causing dysregulation of the HPA-axis, and how environmental and genetic risk factors such as toxins or ApoE4, respectively, turn into disease accelerators under these unnatural conditions. Consequently, the UTAD provides a rational strategy for the prevention of mental decline and a system-biological approach for the causal treatment of AD, which might even be curative if the systemic intervention is initiated early enough in the disease process. Hence an individualized system-biological treatment of patients with early AD is proposed as a test for the validity of UTAD and outlined in this review.

## Background

Alzheimer’s disease (AD) is characterized by impairment of hippocampal episodic memory performance followed by a progressive decline of cognitive and social capabilities. Since AD is the major cause of cognitive decline and no curative drug has been developed, research worldwide is intense and highly competitive. Epidemiological, biochemical, molecular, genetic and animal studies provide different entry points into the complex disease process, which led to different theories about the aetiology of AD. Starting with age as the main cause and primary risk factor, AD is being explained by the oligomeric amyloid beta (Aβ) cascade hypothesis, which includes hyperphosphorylated and dysregulated tau [1], the intoxication hypothesis [2–4], chronic infections [5–7], microbiome composition [8], neuronal insulin resistance [9, 10], physical and functional breakdown of the blood–brain-barrier (BBB) [11, 12], chronic neuroinflammation, due to multiple causes [13], impaired neuronal rejuvenation (NRJ) [14], synaptic failure [15], and a growing list of many others.

All of these theories are more or less deeply embedded in the belief that aging per se is the main etiological cause. In fact, the thought that aging per se is the primary cause of AD is so deeply engrained in our thinking and appears in almost every introduction in any scientific paper about AD to be a compulsory statement, which is rarely challenged. But as I will argue, not only are there a number of serious arguments challenging the “age-is-the-primary-cause-dogma”, ageing as the overarching cause also hinders the development of a “unified theory of AD” (UTAD), which incorporates all key findings including the long list of well-known environmental and behavioural risk factors, hence explaining the aetiology and pathogenesis of this debilitating disease. In fact, the lack of a UTAD continues to limit the development of effective preventive measures and a curative treatment to trial and error. Therapeutic interventions that focus on such singled out mechanisms continue to fail [16]. In addition, prevention trials, which rather base their regimen on the correction of more or less arbitrarily selected risk factors than on a complete theory of AD, were so far also limited in their overall success [17, 18]. In contrast, the proposed UTAD overcomes our concept of age per se as the major cause for AD, and provides an encompassing explanation of the aetiology and pathogenesis of Alzheimer’s. It also allows proposing a number of required individual life changing interventions in order to prevent AD with high probability. In addition, the UTAD might provide the logical framework for a curative regimen, as will be outlined at the end of this review.

I would like to point out that I have termed the proposed theory “UTAD” because it presents a systemic neurobiological framework of how all currently known major behavioural, environmental or genetic risk factors individually or in combinations initiate or accelerate the AD process, despite certain caveats that apply for most if not all theories: Although I tried to be as comprehensive and exhaustive as possible in my search using key words like for instance “AD risk factor”, “factors inhibiting AHN” or “neuroinflammation” in the PubMed database of the National Center for Biotechnology Information, some minor risk factors might have been overlooked, it needs to be seen if they will verify or falsify the UTAD. The same goes for risk factors, which are already acting today but have not been identified yet, or which emanate from future individual lifestyle choices or cultural developments. Conversely, in some categories of risk factors (e.g. environmental toxins and chemicals), I purposely listed only examples, since a comprehensive list (e.g. of all currently known chemicals that negatively influence critical mechanism of the AD process as proposed by the UTAD), would not add to its understanding and therefore go beyond the scope of this review. It is my hope that once the principal concept of the UTAD is accepted, all risk factors that interfere with the neurobiological mechanisms, which, according to the UTAD, are at the centre of AD can be recognized and investigated more efficiently. Last but not least, behavioural risk factors in context of the UTAD might lead to discussions about free will or freedom of action, which would also go beyond the scope of this review.

### Aging is required but not causal for AD

Measurement of insulin resistance of the hippocampal/temporal lobe by positron emission tomography with 2-deoxy-2-[fluorine-18] fluoro-D-glucose integrated with computed tomography (FDG-PET/CT) has become, besides amyloid-PET diagnostics [19] a highly specific and sensitive biomarker for AD, having predictive value even decades before the first clinical symptoms of AD manifest themselves [20]. One may conclude that the development of AD obviously requires time and, therefore, the logical consequence is that the risk of developing AD will increase with age. But correlation does not a priori equal causation. In this case, for a disease requiring time to develop, age might simply be a precondition but not necessarily a cause. If age was indeed the cause of the disease, AD would not only be a natural outcome of human aging but the fight against AD would be a fight against human nature, which is highly difficult to win.

But, fortunately, many lines of evidence disagree with this explanation (for example see [21, 22]). Particularly from a human’s life history point of view, if age per se was indeed the main causative risk factor, why was AD essentially unknown around the beginning of the last century? According to a recent estimate, age would have caused approximately 36 thousand new cases per year in the USA alone, making the disease very common [3]. But a textbook on neurology published in the late 19^th^ century did not even mention an AD-like pathology [23], and in 1906, when Alois Alzheimer first published report about the pathology appeared, he described it as a *peculiar* brain disease [24], suggesting that it has been unknown before. And still in 1938, 32 years later, Alzheimer’s description of amyloid plaques and neurofibrillary tangles as hallmarks of AD brain pathology had not found their way in a comprehensive textbook of pathology [25]. It may be proposed that, at that time, death as a consequence of AD was a very rare event.

AD prevalence was maybe similar rare as in Japan at the mid of last century. A recent study provided significant evidence that not age but rather certain lifestyle factors explain the sevenfold (!) increase in AD prevalence over the last half of the 20th century [26]. This dramatic increase was strongly associated with a change from the traditional Japanese diet/lifestyle towards a Western one, which mainly took place between 1961 and 1985. In particular, besides a large increase in alcohol intake, the consumption of meat and animal products rose by seven- and fourfold, respectively. Animal products and meat are known to increase the risk of AD because they contain compounds such as excess iron (which particularly enhances the risk for ApoE4 carriers, as will be detailed below), advanced glycation end products (AGEs) and arachidonic acid that have been shown to increase oxidative stress and inflammation in the brain (which also will be detailed below). The dietary changes paralleled the dramatic increase in AD prevalence for those people aged 65+ years in Japan, which rose from a low 1 % in 1985 to about 7 % in 2008, with a lag time of about 20 years. This trend in disease prevalence can neither be explained by a change in life expectancy nor by a genetic drift. Such relatively short intervals are rather known for other behavioural diseases like e.g. lung cancer from smoking [27].

According to the author of this groundbreaking study, the AD prevalence rates in Japan may have reached a peak - like in other highly industrialized societies - and will not increase further, as these AD-causing behavioural factors have changed only modestly since 1985. Consequently they one reviewer of the study warns, “unless Japanese people return to the traditional Japanese diet, AD rates in Japan are unlikely to decrease” [28]. A similar increase in AD incidence from low to high rates can be observed in emerging economies. For instance, the age-specific AD incidence rate in rural India was shown to be about four times lower than in the USA [29], its rise rather parallels the rate of economic growth, which strongly influences the lifestyle [30]. Similarly, the prevalence of AD among African-American populations living in the US is several-fold higher when compared to age matched Africans in their homelands [31, 32]. Japanese who migrated to the US and adopted the American way of life increased their AD-risk [33]. Hence, not ethnical origin, but rather the adopted modern lifestyle has an impact on AD risk [34]. In other words, our individual life history might play a decisive role in shaping the AD risk.

According to a reanalysis of the Framingham Heart Study, the incidence of dementia over three decades almost halved [35]: The 5-year age- and sex-adjusted cumulative hazard rates for dementia were 3.6 per 100 persons during the first epoch (late 1970s and early 1980s), 2.8 per 100 persons during the second epoch (late 1980s and early 1990s), 2.2 per 100 persons during the third epoch (late 1990s and early 2000s), and 2.0 per 100 persons during the fourth epoch (late 2000s and early 2010s). Compared to the incidence during the first epoch, the rate declined by 22, 38, and 44 % during the second, third, and fourth epochs, respectively. Again, these data are inconsistent with age per se being the major cause of AD. According to the authors of the study, the key contributing factor to this decline have not been identified, but it is important to note that this positive trend was observed only (!) among persons with at least a high school diploma. For those with lower education, the AD-risk was actually rising by 66 % over the four decades. This indicates that education and/or income, hence socioeconomic factors, might play a pivotal role. But we can certainly rule out age, as low socioeconomic status reduces, rather than increases, lifespan [36]. According to the authors of another study [37], which reported a similar decline, the increased use of antihypertensive, lipid lowering and antidiabetic drugs might have contributed to these positive trends, as well as experiencing less stressful life events like wartimes, which particularly afflicted the older generations in former studies, from which the earliest estimates were derived. Taken together, life choices and life experiences appear to be of etiological significance for AD.

Results from research based on animal models also argues against aging being causative for AD. Laboratory animals are kept under so-called standard housing conditions which represents their “lifestyle”, which is in some aspects quite similar to the life in western societies. This “Western-type-lifestyle” is sedentary, lacks social activities and, comparably, the animals suffer chronic sleep deprivation since they are constrained to an unnatural but standard 12 h dark/light-rhythm. Furthermore, an unnatural ad libitum feeding pattern is also applied routinely to animals in experimental research, leading to a misinterpretation of experimental results, particularly in AD research. Not the animal living in its wild habitat, where e.g. physical activity and intermittent fasting (IMF) is natural, but the sedentary caged animal, which is fed ad libitum, became the standard to which we tend to correlate and to interpret all data down to the cellular and molecular level. In contrast, a more natural housing, named environmental enrichment (EE), which includes social activity in larger enclosures and environmental complexity (e.g. the presence of objects that can be manipulated, structures for climbing or exercise, foraging opportunities, hiding or nesting areas), hence a more natural situation, is regarded as a experimental condition [38]. But it should be vice versa, since the conditions under an EE mimic in important aspects those of our own pre-modern lifestyle, hence the general conditions to which our genetic program is adapted too. The standard housing condition should therefore be regarded as the experimental condition, in which important factors for the physical and mental well-being are eliminated and the consequences for aging and AD being studied. This change of perspective would help to regard AD primarily as a deficiency disease, i.e. a result of discrepancies between behavioural requirements of an organism and its actual lifestyle conditions.

In line with the proposed UTAD, even old mice maintained in EE show a robust fivefold increase in adult hippocampal neurogenesis (AHN) [39], which is an important aspect in regard to AD, as will be outlined in detail below. Furthermore, they exhibit reduced anxiety and depression-like behaviours [40], and show improved memory performance compared to animals maintained under standard housing condition [41]. Therefore, once we start looking at animal models of aging or AD from an evolutionary perspective, experimental deficiencies (under current standard housing conditions) in physical exercise, IMF, social activities or essential nutrients would become obvious causes of AD (see below). For example, one year of IMF prevented cognitive decline even in a triple-transgenic mouse model of AD (which express the APPswe, PS1M146V, and tauP301L mutations [42]), providing evidence that not aging but rather the feeding pattern have long-lasting effects on memory [43]. More examples will follow, which in combination have one important consequence: Most results obtained from animal research (as well as from epidemiological studies) regarding aging and AD need to be reinterpreted from an evolutionary point of view: “Normal” aging under “Western-style” housing is not natural, and the same might apply to AD in humans.

### Lifestyle choices initiate and genetic predispositions accelerate AD pathogenesis

Evolution drives primarily by the selection of advantageous genetic variants. The dramatic trends in AD incidence in industrialized countries over the last century and the similar rise in emerging economies nowadays can therefore not easily be explained by genetic influence. It is more likely that AD follows similar lifestyle changes that parallel the well-known increases of type-2-diabetes, obesity, high blood pressure and arteriosclerosis, all well-known risk-factors for AD [44].

Another rather crude example serving to explain the interplay between a certain lifestyle choice and AD risk and demonstrating interplays with a presumed genetic predisposition comes from the USA. Compared to the general population, players in the national football league (NFL) have a threefold increased risk of developing major depression [45] and those who incurred three or more concussions during their careers had a fivefold higher risk for amnestic mild cognitive impairment (MCI) [46]. According to the authors of the NFL-study, traumatic brain injury is a causative risk factor for AD and other forms of dementia. The average age at AD diagnosis was 53.8 years, which indicates that, in these cases, it is definitely not age which increases AD risk. Several other studies confirmed these finding (for review see [47]): In particular speed players who commonly build up considerable momentum prior to tackling or being tackled, showed even a significant sixfold higher mortality resulting from AD and from amyotrophic lateral sclerosis in comparison with the general US population. Interestingly, ApoE4-carriers (i.e. carriers of a specific genotype variant of the apolipoprotein polymorphism) are more susceptible to concussion-mediated AD-risk, which is in line with the experimental observation that ApoE4, in contrast to ApoE2 and ApoE3, makes particularly the BBB more vulnerable to proinflammatory insults. This alterations lead to an increased neuronal uptake of multiple blood-derived neurotoxic proteins, as well as microvascular cerebral blood flow disturbances. In ApoE4-expressing animal models, those vascular defects precede neuronal dysfunction and can initiate neurodegenerative changes [48].

The ApoE4 polymorphism is known as the most common genetic risk factor for sporadic AD with 15 % Caucasians being carriers. Although ApoE4 is neither necessary nor sufficient for the development of AD, having one or two copies of the ApoE4 allele increases late-onset AD risk about 3- to 12-fold, respectively [49]. Interestingly, ApoE4 is a uniquely human allele and the most ancestral, and its appearance in evolution marks the dramatic increase in the human lifespan (for review see [50]). In contrast, the human ApoE3 allele appears to be neutral regarding AD risk, and its mutation emerged much later in human history; its frequency increased during human evolution with 75 % of Caucasians now being carriers. Why has the ApoE4 allele not been completely replaced by natural selection with the health-beneficial ApoE3 or ApoE2 allele, which even appear to lower AD risk? There are several possible explanations. For instance it is assumed that alleles that are detrimental to health in older age might persist in populations because they confer, by means of antagonistic pleiotropy, some benefit to younger individuals [51]. ApoE is a major supplier of the cholesterol precursor for the production of oestrogen and progesterone. According to one recent study, women who carry at least one ApoE4 allele have significantly higher levels of mean luteal progesterone than women that carry only ApoE2 or ApoeE3 alleles. ApoE4 might therefore be advantageous regarding fertility and therefore in reproductive performance [52]. Another study found an association of ApoE4, when compared to ApoE2 and ApoE3, with good episodic memory and an economic use of memory-related neural resources in young, healthy humans [53], albeit other studies failed to provide evidence for such a benefit [54].

Whatever the advantage of ApoE4 for some might be, we should be aware that all our observations regarding the disadvantages of any genetic variant with respect to AD were observed in human populations (or caged animals) that dramatically changed their lifestyle in recent history. We simply do not know if ApoE4 would be neutral or might even provide a benefit for cognitive health in the elderly, if current behavioural and environmental deficiencies are omitted. Indeed, it is noticeable that particularly the detrimental effects of the ApoE4 polymorphism on cognition may strongly depend on modifiable risk factors (for review see [50]). For example, in some studies, engagement in physical activity seems to reduce AD risk primarily in ApoE4 carriers [55], in line with the “grandmother hypothesis” (see below), which argues that the evolution of the long human lifespan likely required older individuals to maintain high levels of both physical and cognitive health. Like the NFL-example discussed above, recent cohort studies provided evidence that ApoE4 makes us more vulnerable to unhealthy lifestyle choices. In one study, hypercholesterolemia was associated with cognitive impairment mainly among ApoE4 carriers [56]. Hence the advantage in fertility, as has been outlined above, might turn into a disadvantage upon altered behaviour that leads to increased cholesterol levels. In another study, which will be detailed below, ApoE4-subjects adhering to a healthy Mediterranean diet (MeDi) had the greatest benefit regarding brain health when compared to other genotypes [57]. This tendency was confirmed by the finding, that particularly carriers of the ApoE4 allele profited from nutritional consumption of fish, as their AD risk dropped by a factor of two, whereas non-ApoE4-carriers had no such advantage [58]. Furthermore, a recent study provided evidence that ferritin levels in the cerebrospinal fluid (CSF) are negatively associated with cognitive performance in cognitively normal (i.e. averagely mentally declining with age), MCI and AD subjects, and predicted speed of MCI conversion to AD [59]. Interestingly, the elevated CSF-ferritin levels were found to be strongly associated with the ApoE4 genotype. From an evolutionary point of view, ApoE4 carriers might have had an advantage as their brain was provided with sufficient iron in situations of low nutritional iron availability. Since nowadays diets usually contain large amounts of iron-rich animal products (as outlined above for the modern Japanese society), the potential physiological advantages of ApoE4 in iron metabolism might fire back, leading to elevated brain iron, which promotes enhanced production of reactive oxygen species (ROS) and adversely impacts on AD progression.

Hence it is conceivable that the most common genetic risk factor for sporadic AD, namely ApoE4, might be important for brain health under those conditions that prevailed for the longest part of our evolution [50]. Therefore, it might now only be regarded as a risk factor under unhealthy lifestyle choices (like professionally acquired head traumata, favouring life-styles that lead to increased cholesterol levels or iron intake, or low physical activity etc.). The ApoE4-allel might therefore not be a cause of AD but might rather act as an accelerator under such (unhealthy) conditions. Indeed, each ApoE4 allele exerts a dose-related earlier onset of AD [60]. The situation might be similar to the so-called obesity alleles, which simply provide the carrier with a genetic variant that actually improved the fitness of his ancestors by being particular efficient in energy conservation under living conditions, which alternated between IMF and feasts after successful foraging. Nowadays, under lifestyle conditions that provide a steady energy supply under minimal physical expenditure, the same genes are regarded as “disease genes”. The good news, both for carriers of “obesity genes” and particularly of ApoE4 is that they will benefit most from a return to a healthy lifestyle.

Taken together, the aetiology of AD appears not to be different from any other current “culture-borne diseases”, which are caused by well-known deficits in physical exercise, intake of healthy food, intermittent fasting and social activity. The AD epidemic might simply be due to the development of a highly sedentary, overindulgent and occupation-specific lifestyle that largely eliminates extended family bonds and time to relax. In order to prevent and treat AD, we might need a better understanding of how the true causative risk factors interact. But, much more, we need to become more open-minded: We should stop regarding AD as a natural (genetic) disease or a causal consequence of aging and begin to accept that AD is caused by environmental factors and behavioural deficiencies (i.e. the well known risk factors). Consequently, AD should be preventable and, in the early stages of the disease, might even get cured, provided that we (re-) assume a lifestyle that satisfies all essential requirements of our brain, which result from our human’s particular evolutionary life history.

### Evolution of human longevity

We are a product of evolution. Therefore, any explanation of a human ailment like AD must be footed on evolutionary theory if we want to regard the aetiology and pathogenesis as fully understood.

The second law of thermodynamics describes the natural instability of any information content, which includes the one encoded in the DNA in all our cells. Hence life had to overcome a principal obstacle and find a way to preserve genetic information. Nature’s solution was the repetitive duplicating of genetic material (analogous to the need to frequently copy our favourite data stores if we do want to avoid losing their information content). The quality control mechanism after the copying process is selection of those copies that provide their carrier with the ability to efficiently continue the DNA duplication process. This mechanism allowed adaptation to an ever-changing environment and the evolution of quite different reproduction strategies. Bacteria on one end of the spectrum engage in mass production and mass genetic variation to increase the probability of survival of at least a few genetic copies. Humans, on the other end, only have to generate relatively low numbers of descendents in order to survive as a species, since their large brain permits them to engage in complex social collaborations and to use acquired knowledge to enhance the chance of their progeny’s survival: According to large and complete multi-generational demographic records of pre-modern Canada and Finland, for every post-reproductive decade at least two more of their grandchildren reached reproductive age, when compared to families with the grandmother dying earlier [61]. Therefore, the older the grandmother beyond 60, i.e. 70, 80, or 90 (i.e. the age, when age supposedly causes mental decline), the more she was able to increase the number of her grandchildren and the chance of their survival into adulthood, longevity became a selected trait acting by transgenerational generativity. Generativity in a psychosocial sense refers to the human concern for establishing and guiding the next generation.

This particular evolutionary strategy developed early in human history and is known today as the grandmother-hypothesis (GMH) [62]. The GMH explains the evolutionary origin of our exceptional longevity when compared to our genetically closest cousins, the chimpanzees, which die a few years after reaching menopause [63]. The objection to the GMH, that longevity is a rather modern phenomenon, since hunter-gatherer societies have low average life expectancies, can be repudiated, as those low averages reflect high infant mortality and not individual life expectancy. Indeed, in still existing hunter-gatherer societies that are insulated from modern life, about two thirds of those who reach adulthood actually survive up to an age of 70 years, even encountering an 80-year old might be no exception [64]. Reproductive age in hunter-gatherer populations was between 15 and 45 years of age, hence a grandmother became first independent of rearing her own children when her last own child reached the childbearing age of about 15 years. Hence exclusive grand-motherhood, i.e. the years independent of motherhood, started at around 60 years of age and had to last for at least another 15 years, until the first grandchildren of her latest child reached adulthood. Interestingly, the GMH was recently supported by observations of extended Orca whale families [65]. As in humans, female Orcas can live several decades beyond menopause and their reproduction strategy also relies on the use of lifelong acquired knowledge. In particular, the oldest post-menopausal females lead their groups in salmon foraging grounds and organize the hunt especially when salmon abundance is low, which would otherwise reduce reproductive success and drive mortality rates.

Taken together, the GMH contradicts principally with the theories of AD being a general error in our genetic program or simply being caused by aging, since the evolution of longevity as a reproduction strategy requires a brain functioning well particularly at higher age to endow the kinship with the acquired life experience. Furthermore and as the GMH predicts, under conditions closer to those that were active during the long pre-modern area of human development and to which our genetic program is exquisitely adapted, the human brain maintains the lifelong facility to acquire empiric knowledge [66]. One key mechanism is the lifelong growth of our memory store by adding new neurons through AHN, another is the lifelong maintenance of already existing neurons, i.e. neuronal rejuvenation (NRJ) through the mechanism of autophagy and regeneration of subcellular structures and organelles. Both mechanisms, NRJ [67] and AHN [68] appear to be efficiently functioning even at high age, which is in line with GMH (see below), but require certain environmental cues and depend on our lifestyle choices, and if defunct, both are also closely and causally linked to the aetiology of AD, as I will also show below.

### Lifelong neuronal rejuvenation (NRJ)

Mitochondria sustain cellular bioenergetic homeostasis by playing key roles in a broad range of core cellular functions, including energy production, metabolic and calcium signalling, and in a number of biosynthetic pathways [69]. They also perform a variety of functions in processes such as the transduction of metabolic and stress signals and the production of free radicals such as reactive oxygen species (ROS). Originally envisioned as a “necessary evil”, i.e. a by-product of an imperfect oxidative metabolism, ROS are now recognized for having an essential signalling function in cellular physiology [70]. Only in case of mitochondrial damage, excess accumulation of ROS evoke an inflammatory response, which inhibits AHN, but also leads the mitochondrial host to engage the cell death program, which in case of neurons leads to apoptotic neurodegeneration [71]. Hence chronic perturbance of mitochondrial function aggravates the pathogenesis of many neurodegenerative diseases. A complex defence mechanisms against this type of oxidative stress has been resourcefully adapted, which consists of endogenously generated ROS sequestering molecules and enzymes (e.g., α-lipoic acid, super oxide dismutase, glutathione) and many bioactive nutrients (e.g., plant-derived vitamins and polyphenols), which detoxify the harmful effects of ROS, while maintaining their signalling capacity.

In addition, cells like neurons that have a low or no turnover at all only survive up to high age of the individual by maintaining intracellular youth through the process of NRJ [72]. Central to maintaining cellular bioenergetic homeostasis is to sustain a healthy mitochondrial population to ensure that cellular energy demands are met by energy supply [71]. To this end, the exchange of damaged macromolecules and cellular organelles including mitochondria evolved as an active and highly regulated process [73], requiring behavioural cues to initiate. Accordingly, accelerated aging [74], memory decline and AD [75] might be caused by a lack of these cues. Conversely, the accumulation of non-functional macromolecular and damaged mitochondria affect cell function with symptoms arising, when over many years the rate of damage exceeds the rate of repair and turnover [76]. Accordingly, autophagy is important to slow aging, inflammatory processes and cell death [77].

The master regulator/inhibitor of autophagy is the mammalian target of rapamycin (mTOR) [78]. This intracellular kinase functions as a key signalling node that integrates information regarding extracellular growth factor stimulation, nutrient availability and energy supplies [79]. The fungal metabolite rapamycin was accidently found to block mTOR and became not only the eponym of mTOR but also the main molecular tool to dissect mTOR-function. Rapamycin treatment was found to activate autophagy by inhibiting mTOR [80], thereby slowing down both aging and cognitive decline of caged mice [81], suggesting that inefficient autophagy as part of the NRJ-program might be a central element of both processes. Conversely, caging (standard housing) might eliminate an important behavioural cues (like for instance physical activity or intermittent fasting, see below), which leads to unnaturally high activity of mTOR and low activity of peroxisome proliferator-activated receptor γ coactivator 1α (PGC-1α) [82], the master controller of mitochondrogenesis [83] (see below), thereby inhibiting neuron-rejuvenating and protecting autophagy [84]. Hence the observed aging and cognitive decline in murine models of AD might be regarded as artificial and not reflecting the aging process under natural conditions.

Other behavioural cues that inactivate mTOR are, besides physical exercise, which was shown to promote autophagy of defunct organelles and macromolecules in the brain [85], chronic caloric restriction (CCR), which is well known to delay aging and extend life-span in essentially all eukaryotic organism [86]. But is CCR a physiological cue or rather an artefact of experimental research that simulates, to some extent, a more natural dietary pattern, namely intermittent fasting (IMF), which from an evolutionary point of view is more natural (see below)? But IMF is experimentally more labour-intensive than CCR and therefore less well studied. Nevertheless, autophagy and in particular mitophagy was found to being activated by CCR through inhibition of mTOR in essentially all species investigated, ranging from yeast, to flies, worms, fish, rodents and even to rhesus monkeys [87], thereby decelerating mTOR-driven aging [88]. CCR not only extends lifespan, it also protects the central nervous system from neurodegenerative disorders, whereas excessive caloric intake is clearly associated with accelerated aging of the brain and increased the risk of neurodegenerative disorders due to suppressed autophagy [89].

Nevertheless, IMF was shown to create a more robust and steady inhibition of mTOR-accelerated aging and cognitive decline when compared to CCR [90]. This is explained by the fact that the main hormone-like signalling molecules of the metabolic status during IMF, the ketone bodies acetoacetate (AcAc) and D-β-hydroxybutyrate (βOHB), are more efficiently generated during fasting than by CCR [91]. These two respiratory fuels can endogenously be produced by the liver in large quantities (up to 150 g/day) from mobilized fatty acids in a variety of physiological [92] or pathological conditions [93]. In humans, basal serum levels of βOHB are in the low micromolar range, but rise up to several hundred micromole after 12 to 16 h of fasting [92]. Importantly, when blood glucose and insulin are low [94], up to 60 % of the brain energy needs can be derived from ketone bodies, replacing glucose as its primary fuel [95]. Similar high levels of up to 1 to 2 millimole βOHB are reached after prolonged endurance exercise [96]. A physiologically relevant increase in ketone body production is already achieved by fasting overnight, which can even be enhanced if we are physically active before breaking the fasting in the morning [97]. This most likely mimics the situation that faced our foraging ancestors who went out for hunting or gathering food with their stomachs empty.

Since neither long- nor medium-chain saturated fatty acids can pass the BBB, only their transformation into ketone bodies allows our energy-demanding brain to access the largest energy store, our adipose tissue. In fact, ketone body production reduces glucose requirement and preserves gluconeogenic protein stores during fasting, which enables a profound increase in the capacity for survival [98]. Interestingly and again in line with the GMH, elderly generate ketone bodies at least as efficient as younger adults during IMF [99] and the metabolic response to a ketogenic diet appears also to be unaffected by aging [100].

As hinted at above, the observation that IMF is superior to CCR makes also a lot of sense from an evolutionary perspective, as not chronic starvation but rather periodic alteration between fasting and intake of high-caloric meals after successful foraging was ancient normality. Importantly, recent evidence suggests that our phylogenetically conserved genetic program uses the metabolic changes that originate from intermittent fasting (IMF) as a behavioural cue of for the initiation of subcellular renewal [101]. This is a good thing, since in order to maintain cellular youth, we do not have to starve by CCR. It is sufficient to alternate phases of fasting, which just need to be sufficiently long to induce ketone body production (for instance 12 h overnight) and phases of eating, in which the total energy demand of our body can be met. In contrast, current normality consists of constant feeding pattern, which results in permanent high mTOR activity (and low PGC-1α-levels, see below), which suppresses cellular rejuvenation. A sedentary lifestyle aggravates this pro-aging effect, whereas prolonged physical exercise reduces mTOR-activity [102], possibly also by increasing ketone body production.

Taken together, inhibiting mTOR by being physically active and by IMF is a natural way to stay healthy. But there are always hopes that instead we can put a healthy lifestyle in a pill: Rapamycin is an approved immunosuppressant in humans used to prevent rejection in organ transplantation and meanwhile clinical trials are under way to test efficacy in AD (for review see [103]). But given the fact that IMF, a nutrient-rich diet and regular aerobic exercise and sleep have more beneficiary functions than just inhibiting mTOR, it is questionable if long-term health can be achieved by a drug without a lifestyle change. For instance, many of the well known beneficiary effects of intermittent fasting (IMF) as well as physical exercise are mediated by the upregulation of PGC-1α. But activation of PGC-1α also reduces neuroinflammation [66], for instance by inducing the expression of ROS-detoxifying enzymes [104]. Hence two potential causes of aging, chronic mitochondrial dysfunction and neuroinflammation (see below), can be explained by reduced levels of PGC-1α [105], and might originate from either a lack in physical exercise, IMF, or both; or from many other behavioural deficiencies, which will be discussed in detail below. Hence I doubt that behavioural deficiencies can be compensated by providing a single drug.

In summary, behaviourally suppressed cellular rejuvenation affects all organ systems and leads to frailty when we age. But we should not fall into the trap of blaming frailty or aging per se as the primary cause of physical and in particular cognitive decline [106]. “Normality”, as we usually define and see it, might be misleading, since it only reflects a statistical mean of current behaviour and its consequences (like e.g. the average decline of cognitive abilities with age). In fact, we prolong our lifespan despite unhealthy aging because we nowadays benefit from intense medical care [107]. But the ability to reversing the trend is in our own hands, thanks to the genetic program that evolved with our ancestral grandmothers.

### Physiological role of amyloid beta (Aβ)

The hippocampus complex (HC), i.e. the archecortical hippocampus and entorhinal cortex, is the central organ for remembering personal life experiences [108]. Since every moment in life is unique and cannot be repeated like vocabulary, this old part of our brain has maintained the ability to learn essential information instantaneously. This capability is a prerequisite for spatial navigation in new terrain like during gathering and hunting, as well as social learning from personal interactions, narrations and is even important when remembering long-term ones’ own thoughts. As an indicator of their potential significance for survival and reproduction, the HC selects experiences for memorization by their emotional effect. (Unfortunately, vocabulary is rarely exiting enough to be fast remembered). The HC thereby memorizes at least three interrelated aspects of the life episode: what happened (content information), where it happened (spatial information) and when it happened (temporal information). As the HC has a limited memory capacity, the daily acquired content information is stored only transiently. In order to remember important (emotionally exiting) life events lifelong, the content information is being consolidated in the neocortex during the slow-wave sleep (SWS) phases in the early part of the following nocturnal sleep [109]. In the second part of nocturnal sleep, the new memory is being cross-linking with former experiences during the rapid eye movement (REM) phases, a process thought to inspire insight while we dream [110]. The spatiotemporal information, i.e. the contextual “where and when” of the memorized event, is maintained long-term in the dentate gyrus (DG) network of the hippocampus and used as an index for retrieval (reactivation) of the neocortical memory traces (see below).

Hippocampal fast learning relies on the mechanisms of synaptic long-term potentiation (LTP) using glutamate as a key neurotransmitter. Glutamate alters the strength (weight) of specific synaptic connections thereby creating a new hippocampal memory trace. Any increase of neuronal activity induces concomitantly an increase of β-amyloid (Aβ) production and release on the same synapses, where Aβ plays an important role in learning memory formation [111]. Aβ is mainly secreted in monomeric form with 40 amino acids (Aβ40), which has a slight tendency to aggregate. Conversely, Aβ42 is produced in low quantities and is more prone to the formation of oligomers, protofibrils and fibrils. These aggregates represent the main form of Aβ contained in AD brain plaques [112]. This observation and the fact that conglomerating Aβ42 is produced in higher quantities by certain AD-mutations [113], has led to ascribe pathological effects to the oligomers and the physiologic properties of Aβ almost solely to monomers. It was also the foundation of the amyloid cascade hypothesis.

However, a certain degree of oligomerization is likely to occur even at low Aβ concentrations, hence the native state characteristics of the peptide and in which it exerts its multiple functions are difficult to elucidate [114], particularly, as the oligomeric states of Aβ vary [115]. In any case, the concentration of soluble Aβ in the normal healthy brain has been estimated in the picomolar range with species ranging from monomers to higher oligomers [116]. One important physiological function of Aβ might be as a regulator of glutamate release probability [117], whereby the activity-dependent modulation of Aβ production acts as a negative feedback regulator of synaptic plasticity [118]. It has been shown, that at lower, picomolar concentrations, Aβ increase LTP, whereas at higher, nanomolar concentrations, Aβ leads to a reduction of potentiation [119]. This is in line with the proposed mode of action, in that Aβ, depending on the dose, either acts as an agonist, supporting the fast formation of new memories, or antagonist, preserving new memory traces from being overridden by subsequent events that are to be memorized [120].

If this negative feedback loop gets compromised, either to much Aβ is being produced or to little might get cleared. Subsequently, higher concentrations of soluble oligomeric Aβ form and exert synaptotoxic effects, even without plaque formation [121]. This might explain the relative high proportion of symptomatic patients with clinical signs of early AD and a negative scan for Aβ-depositions, the so-called suspected non-amyloid pathology [122]. With the realization that soluble Aβ oligomere distribution correlates better with cognitive decline in AD than the prototypical amyloid plaques [123], the former amyloid cascade hypotheses was replaced by the oligomeric cascade hypothesis [124].

The build-up of high amounts of oligomeric Aβ induces neuroinflammation, causes oxidative damage and negatively influences multiple signal transduction events including the activation of glycogen synthase kinase-3β (GSK-3β) [125], a pivotal kinase in AD but also for memory consolidation, which limits its use as a target for AD therapy. For instance, activation of GSK-3β inhibits AHN and promotes neuroinflammation and apoptosis (for review see [126]). Furthermore, its upregulation leads to phosphorylation of the amyloid precursor protein (APP) and tau, both associated with the pathological processes that lead to the hallmarks of Alzheimer’s disease (AD), i.e. Aβ-plaques and neurofibrillary tangles, respectively [127]. GSK-3β activity thereby shifts tau, a stabilizer of microtubules, to a hyperphosphorylated non-functioning state (p-tau) [128]. Tau pathology is progressive and detrimental to affected neurons via both, loss of tau function [129] and gain of toxic function from pathologic p-tau aggregates [130]. Hence not only high concentrations of oligomeric Aβ but maybe rather dysfunctional p-tau might be regarded as a key driver of AD progression, which is corroborated by the fact that p-tau deposition more closely correlates with disease stage [131].

But are Aβ or p-tau the initiators of consequences of AD pathogenesis? This question has been debated for a long time. For example, the proponents of the amyloid cascade hypothesis blamed primarily age as the causative factor for Aβ-plaque formation and the focus of pharmacological efforts were therefore driven towards the elimination of amyloid plaques. Ironically, not the insoluble plaques but, as mentioned above, rather the excess of soluble oligomers were recently shown to be harmful [132]. But even the removal of Aβ-oligomers by current pharmacological strategies that evolved from the newly proposed oligomeric cascade hypothesis might turn out to be harmful. Not only because the primary causes of AD remain ignored (see below), but also, because Aβ is in fact required for synaptic plasticity. For instance, the healthy physiological function in LTP and memory appears to depend on a delicate balance between the secreted Aβ monomer (in different length and concentrations) and multiple oligomeric states (for review see [133]). This caveat might explain why in experimental animal models for AD, the removal of Aβ by different antibodies against Aβ, which have also used in study settings for human treatment, was not only ineffective at repairing neuronal dysfunction, but also caused detrimental cortical hyperactivity [134]. It has been concluded that this unexpected finding provides a possible cellular explanation for the lack of cognitive improvement by immunotherapy in human studies [135, 136]. In other words, the sole attack against Aβ interferes with neuronal homeostasis, causing a further impoverishment of learning and memory.

Hence the current key strategy to cure or AD at least to delay disease progression might even worsen the status of the patients. As Puzzo et al. recently put it [133]: “the vision of Aβ exclusively as a”bad’ protein has probably prevented us to focus on other important aspects of the disease. “The authors point out further that”Aβ is already present inside neurons in infant brains, and even increases up to 8 years of age, a period of high brain plasticity, when about half of the neurons are Aβ-immunopositive. In adulthood, Aβ is present in the major part of the neurons whereas in aged people there is a 20 % reduction. “Furthermore, for the deposition of Aβ in the brain, only Aβ seeds are critical but not the age of animal models [22]. But if not age, what other factors might lead to Aβ seed formation? Only the answer to this critical question will open a pathway to AD prevention strategies and an AD therapy that is truly causal. But it becomes more and more clear that a rise of Aβ-concentrations is neither inevitable nor a natural consequence of aging. Rather other factors are playing a role and, intriguingly, most of them can be modified in various ways, one of them being sleep.

### Memory growth during sleep

One key function of sleep is the consolidation of new hippocampal memory traces in the neocortex for long-term storage and gaining lifelong experience by integration them into the existing body of knowledge. According to the synaptic-homeostasis hypothesis, this is achieved by repetition of the memory content during SWS but also by the differential renormalization of synaptic weights, which includes selective long-term depression, essentially the reversal of the effects of LTP [137]. Correspondingly, in order to enable hippocampal encoding of new memories during the following wake-phase, Aβ levels, which accumulated during the information collection phase the previous day, needs to be reduced in order to reactivate the full potential of LTP. Hence Aβ-clearance might have developed as another active function of sleep and is accomplished via an enlargement of the interstitial space, which results in a striking increase in convective exchange of interstitial fluid with cerebrospinal fluid and export of Aβ across the BBB [138]. This restorative action regarding memory function also reduces the risk of the concentration-dependent aggregation of larger quantities of potentially oligomeric synaptotoxic Aβ [139]. It is therefore an important finding that lack of sleep in mice and humans increases Aβ concentration above the critical threshold where massive oligomeric aggregation occurs [140, 141].

Another important function of sleep is to provide the temporal space for AHN. During sleep, cortisol, which in high concentrations inhibits AHN, is downregulated, whereas insulin-like growth factor 1 (IGF-1), growth hormone (GH), melatonin as well as BDNF, which all promote AHN, are upregulated. Hence prolonged sleep deprivation is detrimental to AHN [142] and thereby decreases the number of possible distinct codes (indexes) that boost hippocampal memory capacity and performance (see below) [143]. Similarly, posttraining ablation of adult-born neurons was shown to destroy previously acquired memories [144]. New granular cells in the DG are particularly important in differentiating former from similar but novel experiences by remembering their spatiotemporal context, and thereby reducing interference of new with former memories [145, 146]. Furthermore, in order to remember, i.e. to access, retrieve the respective event-specific neocortically distributed stored memory traces and to reconstruct the contextual experience, the hippocampal spatiotemporal information of an remembered event is required [147], as originally outlined by the hippocampal memory index theory (HMIT). According to the HMIT, hippocampal–cortical system consolidation of remote memories requires the maintenance of hippocampal indexes [148]. Hence, we remember episodes of our life by the spatiotemporal context stored by the new neurons generated by AHN [144]. Therefore, remote memories are best maintained by the lifelong creation of new adult-born DG-neurons [149]. The expansion of the spatiotemporal memory capacity thereby becomes also a prerequisite for the continuous expansion of autobiographic memory [150]. This explains, why a disturbed AHN not only causes the hippocampal archive of indexes that link to episodic neocortical engrams running out of storage capacity, but also, why it causes discrimination errors (interferences) between former and new experiences which leads to an overgeneralization of fear and sustained posttraumatic stress [151]. Recently it was shown that in transgenic mouse models of early AD, direct optogenetic activation of hippocampal memory engram (index) cells results in memory retrieval despite the fact that these mice are amnesic in long-term memory tests when natural recall cues are used, which reveals a retrieval, rather than a storage impairment [152]. Interestingly, optogenetic induction of LTP at perforant path synapses of dentate gyrus engram cells restores both “age-dependent” spine density and long-term memory of the caged animals, explaining, why for instance social activity prevents memory decline in an AD model of environmental enrichment, and, thus, not age, but rather an unnatural lifestyle causes AD in these models [153], as will be outlined in more detail below.

The reduced interference of new with former experiences in an expanding archive of indexes explains comprehensively the importance of AHN and how this process promotes persistence [154], as well as precision of contextual memory [155]. In order to remember efficiently, adult-born hippocampal brain cells show a lower threshold for LTP, i.e. learning, while in turn, LTP up-regulates dentritic spine density, with the largest changes occurring during the early phase in their maturation, when they begin to form synapses with the existing circuitry [156]. Via these mechanisms maturating young adult-born DG-cells are being converted into high information neurons specifically by the newly experienced events. Interestingly, conditions, like for example voluntary exercise, that are required to stimulate AHN, also increase spine density in the hippocampus, leading to increased synapse formation and decreased synapse elimination as well as increased survival rates of the maturing neurons [157]. Following this observation, some researchers suggested the hypothesis, that a primary function of AHN involves the production not only of new neurons per se, but of new neurons with the synaptic properties of relatively young neurons [158]. As this mechanism provides for a turnover of almost or 2 % of the cells per year in the DG, AHN has the potential to keep this brain region, critical for acquiring novel experiences, young even at old age [159]. But only, in line with the UTAD, if we provide the behavioural cues for the initiation of AHN as well as the maturation and integration of the new DG-neurons (see below).

### AHN regulates mood and has antidepressant properties, implications for AD

It is of interest that in AD, the perirhinal (PRH) and lateral entorhinal cortex (LEC) of the HC are among the first cortical regions to be affected [160, 161]. Both regions were found to provide the most critical content information (i.e. “what is happening”) to the newborn DG-neurons via the perforant pathway projections (for review see [162]). In addition, back-projecting signals from the CA3 region of the hippocampus, which receives direct signals from the medial entorhinal cortex (MEC) provide the required spatiotemporal information (i.e. “where and when is it happening”) to the newborn DG-neurons [163]. Both inputs appear to be required for event or pattern separation [164]. The findings that hippocampus degeneration is one of the most prominent and earliest characteristics of AD [165] with the perforant pathway being one of the very first structures to suffer damage [166], and that intra-hippocampal white matter lesion load is strongly associated with progressive MCI [167], supports the hypothesis that chronically disturbed AHN might play a etiological key role in AD pathogenesis. This hypothesis is further supported by the evidence that all behavioural deficiencies as well as environmental toxins are known to impair ADH and to increase the risk of AD (see below). Therefore, a non-productive AHN might connect not only histopathologically but also functionally with the aetiology of AD. Hence the understanding of this connection and the requirements for a productive AHN might help to develop a new understanding of AD and a causal strategy for prevention and therapeutic intervention.

Besides being required for spatial navigation, episodic learning and memory retrieval [168], new neurons generated by AHN also regulate mood and in particular psychological resilience (resistance to stress) by controlling the HPA-axis either directly [169] or indirectly [170, 171]. If AHN is disturbed, corticosterone levels are slower to recover to baseline following moderate stress, and the HPA-axis is less suppressed by dexamethasone, showing impaired HPA-axis feedback, in older mice that experimentally lack new adult-born DG-neurons. This decisive involvement of active AHN in regulating psychological resilience is further evidenced by the observation that antidepressants like fluoxetine, member of the selective serotonin reuptake inhibitor (SSRI) class, were shown to require an intact hippocampal neurogenic niche and a productive AHN to exert their antidepressant effect [172]. This antidepressant mechanism was identified in rodents and confirmed in non-human primates [173]. Similarly, a recent human study reported a significant increase in hippocampal volume due to antidepressant treatment [174]. Conversely, impairment of AHN leads to anxiety (inhibition of a novelty-seeking behaviour), major depression [175] and chronically elevated cortisol, all three distinctive warning signs of early AD and potential causative risk factors [176, 177]. Interestingly, HPA-axis dysregulation was shown to occur at least as early as at the MCI stage of AD and to accelerate disease progression [178]. Furthermore, a reanalysis of the data of the Framingham Heart study found that depression at old age develops prior to cognitive decline and was a significant risk factor for dementia and AD [179].

Another hint that disturbed HPA-axis regulation caused by a chronically disturbed AHN might lead to AD, is the finding that early-stage AD patients consistently show increased basal plasma cortisol levels [180] and also decreased sensitivity to low-dose DEX suppression [181]. For this reason, cortisol measurements have been suggested as a reliable AD biomarker [182]. Further evidence, that one the earliest pathogenic events in AD might be caused by an inefficient AHN came from magnetic resonance imaging (MRI) studies: hippocampal atrophy more so than Aβ measures predicted the time-to-progression from MCI to AD [183].

Further support for the hypothesis that HPA axis dysregulation might be an important causal contributor to memory decline in AD comes from the recent prospective Washington/Hamilton Heights Inwood Columbia Aging Project, whose findings clearly revealed that depressive symptoms in late life precede memory decline, but not vice versa [184]. In addition, higher scores on the depression measures predicted steeper cognitive decline even among individuals whose cognition was not pathologically altered at baseline, independent of age, sex, education and illness burden including vascular disease. This observation is in line with evidence of former studies that showed that co-morbidity with depression is associated with a greater extent and progression of AD pathology, such as increased neurofibrillary tangle load [185] and faster rate of cognitive decline [186].

### Chronic HPA axis dysregulation promotes AD pathology

Acute stress leads to immediate physiological changes that promote an increase in sensory input. While heightened senses in life threatening situations are important for survival, these alterations harbour the risk of information overload and hippocampal neurotoxicity due to excessive excitatory glutamate release [187, 188]. Hence it appears to be a useful adaptation that stress-induced HPA-axis upregulation concomitantly increases the production of hippocampal Aβ [189], which acts, as discussed above, as regulator of glutamate release. Furthermore, Aβ monomers activate the phosphatidylinositol-3-kinase pathway, thereby inhibiting apoptosis and protecting neurons from excitotoxic death [190].,Monomeric Aβ was also shown to be neuroprotective by increasing neuronal activity-dependent glucose uptake through activation of the type-1 IGF-1-receptor, which is vital for maintaining neuronal glucose homeostasis [191]. In addition, monomeric Aβ protects neurons against oxidative stress in situations of high energy metabolism [192, 193].

How does activation of the HPA axis increase synaptic Aβ release? One recently identified mechanism is up-regulation of amylogenic γ-secretase activity by the stress-response neuropeptide corticotropin releasing factor (CRF) [194]. Moreover, experimental administration of glucocorticoids was found to increase hippocampal Aβ concentrations in aged nonhuman primates due to reduced production of insulin-degrading enzyme, which is known to degrade excess Aβ [195]. Further in vitro and in vivo experiments provided evidence that glucocorticoid treatment also enhances Aβ production by increasing steady-state levels of amyloid precursor protein (APP) and Aβ cleaving enzymes [196]. Interestingly, these experiments also showed that glucocorticoids augment tau accumulation, indicating that this hormone, if chronically active, might accelerate the development of neurofibrillary tangles. The authors conclude that notoriously high levels of glucocorticoids, found in AD patients, might not merely be a consequence of the disease process but rather play a central role in the development and progression of AD. In support of this idea, chronic hypersecretion of cortisol in patients with lifetime major depression results in significant accumulation of Aβ in the brain, even in the absence of MCI or AD symptoms [197].

Taken together, while enhanced release of hippocampal Aβ in acute stress situations appears to be a useful neuroprotective mechanism, this up-regulation might turn into a problem, when chronic stress or a chronically disturbed HPA axis regulation due to an impaired AHN leads to reduced psychological resilience (weakened stress resistance) and chronic cortisol hypersecretion. Hence the question arises, how our organism sustains a lifelong productive AHN and functioning HPA axis regulation.

### Key requirements for productive AHN and the law of the minimum (LOM)

Since its discovery in animals and particularly in humans, numerous studies revealed that AHN is a highly regulated phenomenon, which is under the control of local factors (the neurogenic niche), cytokines, growth factors and many hormones [198, 199], most of which are directly or indirectly controlled by behavioural or environmental cues. The study of food-storing [200] and song-learning birds [201] revealed the phenomenon of seasonal growth of functionally hippocampus-like brain structures and that AHN and behaviour (e.g. food-storing or song-learning) are dynamically interrelated [202]. This type of research has been extended to mammals, in which it was shown that the hippocampal size for example in kangaroo rats (Dipodomys) tightly depends on their natural space-use pattern [203], or that photoperiodic organisms like white-footed mice (Peromyscus leucopus) monitor environmental day length to engage in seasonally appropriate adaptions in physiology and behaviour, which is preceded by adjustment in brain volume via AHN [204]. This tight environmental and behavioural control of AHN makes sense from an evolutionary point of view, since brain size corresponds to energy expenditure. Hence the size of the hippocampus either grows or shrinks, depending on the individual’s vital need for memory capacity. Since our identity and the principles of the GMH depend on our lifelong ability to remember, AHN in humans has the propensity not only for seasonal but rather lifelong growth.

What are the environmental and behavioural cues and key requirements for lifelong productive AHN? Since mankind survived for the largest part of its history as hunter-gatherers, it is reasonable to assume that the complex regulation of a productive AHN was strongly adapted to the principle conditions, under which mankind survived for hundreds of thousands of years. From an evolutionary perspective, survival of the tribe depended on memorizing the foraging ground under conditions of physical activity and hunger until hunting or gathering was successful, whereby social bonding (hunting in groups) and learning from elders (for instance hunting strategies) were critically important. It is therefore of no surprise that social and physical activity, daily new challenges that provide eustress, IMF interrupted by ingestion of fresh and highly variable food, rich in essential nutrients, are all positive regulators of AHN (see below). Hence the basic requirements for a productive neurogenesis were naturally provided for by the pre-modern way of life. In contrast and as will be detailed below, lack of physical exercise, loneliness (actual or perceived), chronic distress or an unhealthy Western diet (WeDi) combined with a constant (ad libitum) eating pattern are negative regulators of AHN, with the detrimental consequences outlined above.

Starting from the proliferation of the neuronal stem cells, expansion of progenitor cells, followed by their growth and maturation (branching and synaptogenesis) to the final integration into the existing DG network takes about three month, maybe even longer [198, 205]. Besides the fact, that proliferation itself is tightly controlled, requiring both activation and removal from inhibitory signals, only a single digit percentage of newborn progenitor cells become mature, integrated DG-neurons, with most of the cells dying early [206, 207]. AHN requires at each step specific environmental cues (usually transmitted through hormones), but also specific brain building-blocks (essential polyunsaturated fatty acids, cholesterol etc.), social input (new memorable life experiences) and energy. Since each of these factors might individually limit the AHN, the “law of the minimum” applies. This is an important aspect of the proposed UTAD, as this “law” allows us to understand, how AD can be effectively prevented and treated, and why monotherapeutic interventions might continue to fail.

The principle that productive growth is restricted by limiting factors was first formulated in agricultural science by Carl Sprengel in 1828 [208] and was extended and popularized by Justus von Liebig as the law of the minimum (LOM) in 1840 (for references and history of the Sprengel-Liebig LOM see [209]). The LOM states that growth is controlled not by the total amount of resources available, but by the scarcest resource. This is a very important notion if we regard AHN for what it is: a growth process. The LOM explains how a deficiency in any essential growth or maturation factor cannot, by definition (since being essential), be compensated by another. Hence the lack of the respective behavioural or environmental cues or other essential growth factors, which limits the proliferation, growth or maturation of new DG-neurons, must inevitably lead to a disturbed AHN, with the consequences of cortisol hypersecretion, depression and an increased risk of developing AD.

**Fig. 1:**
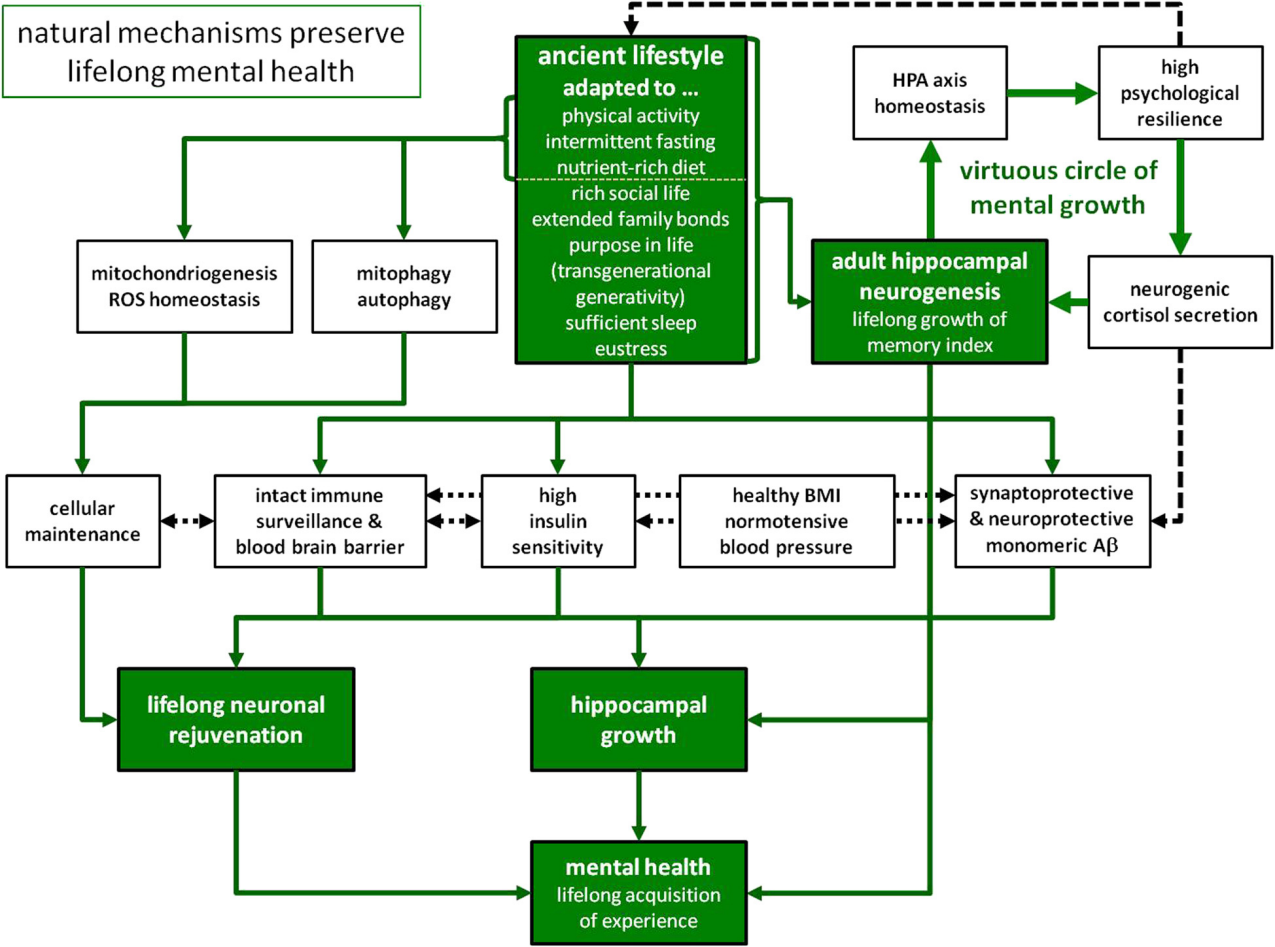
“Natural mechanisms preserve lifelong mental health”. Our genetic program is well adapted to the lifestyle conditions that dominated for the largest part of humans’ life history. Ancient lifestyle was marked by extensive and daily physical activity, alternated phases of fasting and a nutrient-rich diet, sufficient sleep and eustress. Furthermore, survival was critically dependent on extended family bonds, hence went along with a rich social life. The exceptional long postmenopausal period served the purpose of transgenerational generativity, which, according to the grandmother hypothesis, provides the main current explanation for humans’ longevity. Therefore, under these natural conditions, all key physiological systems (immune and cardiovascular function, energy metabolism etc.) positively interact and thereby support key mechanisms like neuronal rejuvenation and adult hippocampal neurogenesis in order to allow the lifelong acquisition of knowledge and to preserve mental health up to high age. For details, please refer to the main text

**Fig. 2:**
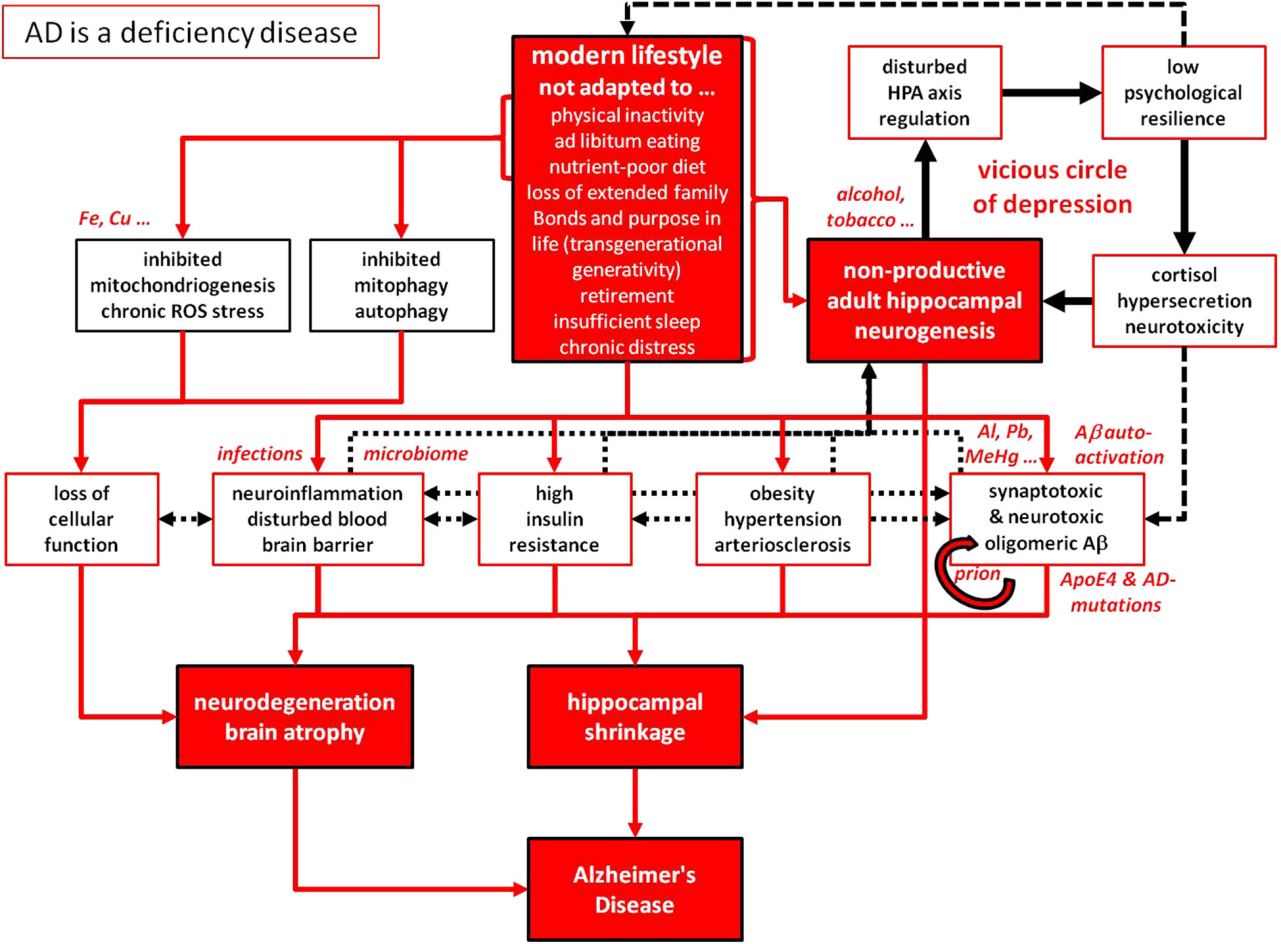
“AD is a deficiency disease”. Our genetic program is not adapted to the fast and very recent changes that define our modern lifestyle, which connotes individual combinations of physical inactivity, an ad libitum eating pattern of a nutrient-poor diet, chronic distress emanating from the demands of a highly competitive labour situation, which often goes along with a loss of extended family bonds. Furthermore and under such conditions, the concept of retirement, a late invention in humans’ cultural history, counteracts the main purpose in late life from an evolutionary point of view : A lack in transgenerational generativity leads to a devastating lack of purpose in life. Deficiencies in essential requirements for mental health cannot, by definition, be compensated by our genetic program. Consequently, as defined by the law of the minimum, individual deficits hamper neuronal rejuvenation, and in particular hinder productive AHN. As the neuronal correlate of depression, the disturbed HPA axis regulation and cortisol hypersecretion as well as other pathophysiological consequences (neuroinflammation and breakdown of the blood brain barrier, insulin resistance, hypertension and arteriosclerosis) emanate from these lifestyle-derived deficits and lead to an accumulation neurotoxic Aβ, hippocampal shrinkage in particular, and brain atrophy in general, hence the well-known hallmarks of AD. Under these conditions of behavioural deficiencies, environmental toxins, chronic infections and genetic predisposition accelerate AD progression. The indicated interactions between the different pathological processes activate a multitude of vicious cycles that make the AD-process a runaway phenomenon, which can only be stopped and reversed to the situation depicted in Fig. 1 by a systems biological approach, which is outlined in the main text and schematically presented in Fig. 3

Epidemiological studies have identified many risk factors for AD as well as for major depression, and many of those are limiting and essential factors for AHN according to the LOM, and most if not all of them are behavioural and therefore modifiable (see also Fig. 1 “requirements” for, and Fig. 2 “deficits” inhibiting a productive AHN). Since the dynamics of hippocampal growth include the hippocampus’s potential to shrink, each factor missing not only hinders a productive AHN, but also leads to a reduced hippocampal size and increased risk for depression and AD. One important prediction emanates from the LOM when applied to a potential multi-causal disease like AD, where individual lifestyles usually lead to several deficiencies, intervention trials, which arbitrarily try to eliminate one or only a limited number of such deficits (i.e. risk factors), can have only weak effects, because only a fraction of the study population will have a deficit only (!) in this (or these) particular factor(s) and therefore benefits from the respective intervention. Following is a commented list of requirements for a productive AHN, many of which are usually in deficit in economically advanced societies (see also Figs. 1 and 2):

Moderate but extended physical activity increases brain volume even in aging humans [210]. This makes a lot of sense from an evolutionary point of view: Physical activity was a prerequisite for survival in pre-modern societies and hormonal signals from the working body are used as a mechanism to enhance not only fitness but and also autobiographic memory capacity (see below). In a sense, being physically active signals to the brain that new experiences are to be expected: The further one walks the more one will experience and the larger one’s hippocampal memory index has to grow. Nowadays, we have the choice to run or not to run, and most of us embrace effort-sparing technologies and lead sedentary lifestyles. The rise of physical inactivity contributes enormously to the overall quality of life by diminishing strength, the ability to perform daily chores and social interactions, mobility and cognitive performance.

In a well-controlled randomized study, 120 older adults without dementia were randomly assigned either to an aerobic exercise group (*n* = 60, average age 67, 6 years) or to a stretching control group (*n* = 60, average age 65,5 years) [211]. The groups did not differ in hippocampal baseline volume as measured by MRI. Besides being healthy, a prerequisite to be part of the program was a sedentary lifestyle, defined as being physically active for 30 min or less daily in the last 6 month before the intervention. After an initial 6 weeks training phase, the aerobic walking group reached a level of 40 min walking daily with a target heart-rate zone of 60–75 of the maximum heart rate reserve, the stretching and toning control group exercised with dumbbells or resistance bands, trained to improve balance including a yoga sequence. After the one-year intervention, the aerobic exercise group showed an increase in hippocampal volume of about 2 %, counteracting the usual hippocampal shrinkage in volume of 1–2 % annually in older adults even without dementia [212]. Such a loss of volume increases the risk for developing cognitive impairment as outlined above. The stretching control group fitted this pattern of age-related loss and demonstrated a 1.4 % decline in volume over the one-year interval, indicating that aerobic exercise is a requirement for brain health at old age, and stretching is not sufficient. That long-term mild aerobic exercise is superior in enhancing AHN has also been shown in rodents [213]. In fact, short bouts of even intense physical activity had no positive effect on AHN [214].

Interestingly, the individual improvements in aerobic fitness of members in the walking group strongly correlated with the increase in hippocampal volume. Growth occurred mainly in the anterior section, which includes the DG. This is a significant finding as cells in the anterior hippocampus mediates acquisition of spatial memory as well as verbal memory performance [215] and is more prone to age-related atrophy compared with the posterior hippocampus [216]. Hence the positive effect was directly and significantly related to improvements in memory performance, indicating that increases in hippocampal volume due to aerobic exercise augments memory function even in late adulthood.

Changes in physical activity have also important consequences for health in general and lifespan. In line with the GMH, an unnatural sedentary behaviour in 70-year-old men was shown to reduce the probability of survival up to the age of 90 from 54 to 44 % [217]. Physical inactivity leads to many so-called “lifestyle diseases”, which are sometimes rather inappropriately named diseases of longevity, as if age were the cause rather than a chronically unhealthy lifestyle [218]. Declining levels of expression of neurotrophic factors level such as BDNF, nerve growth factor (NGF) and glial cell-derived neurotrophic factor (GDNF) are strongly implicated in aging and AD [219]. But correlation with age does not equal causality [220, 221]. Rather, all these hormonal factors are positively regulated by exercise, explaining the accelerated brain aging is rather caused by a sedentary lifestyle in the elderly [222], and therefore appears to have little to do with aging per se.

This direction of causality, i.e. from lifestyle to brain “degeneration” over the years, was tested in animal experiments. In one setting, downregulation of GDNF in sedentary triple-transgenic AD mice was reversed by voluntary exercise and improved synaptic functioning [223]. This particular AD-model triple-transgenic model (3 × Tg-AD) harbours the PS1M146V (a presenilin 1 mutant), APPSwe (a β-amyloid precursor protein mutant), and tauP301L (a p-tau mutant) transgenes and was specifically developed for evaluating the impact potential AD therapeutics of both amyloid plaque and p-tau tangle pathology on synaptic function [42]. In another experiment, NGF was also up-regulated by physical exercise and shown to stimulate AHN [224]. Furthermore, BDNF was shown to promote differentiation and maturation of adult-born DG-neurons [225] and was being released from the hippocampus upon exercise [226].

In the above outlined aerobic walking intervention study, increased hippocampal volume in the exercise group was also associated with increased levels of serum BDNF, which were shown, at least in rats and pig, to parallel hippocampal BDNF concentrations [227]. The observation that BDNF secretion in the hippocampus can be induced by exercise is an important finding as this member of the nerve growth factor family was found to be reduced in the hippocampus and the temporal cortex of AD patients [228]. Another intervention study in humans provided further evidence that hippocampal volume loss, which was significantly and directly associated with poor memory performance, could be counteracted by moderate-intensity exercise [229]. Interestingly, both exercise-induced erythropoietin (EPO) and vascular endothelial growth factor (VEGF), released in reaction to general or local decrease in oxygen concentration, respectively, enhance AHN. VEGF was even shown to be necessary for exercise-induced AHN [230, 231]. EPO was found to enhance hippocampus-dependent memory by modulating plasticity, synaptic connectivity and activity of memory-related neuronal networks [232]. It also activates AHN [233, 234] and protects hippocampal neurons through increasing the expression of BDNF [235].

The importance of physical activity in the prevention of chronic diseases and in particular AD is also exemplified by BDNF’s ability to stimulate both, PGC-1α-dependent mitochondrial biogenesis in hippocampal neurons and promotion of synapse formation and maintenance [236]. Activating PGC-1α through exercise also plays a key role in inhibiting AD-driving neuroinflammation [237]. The significance of physical exercise for the brain’s health is further substantiated by the fact that evermore hormonal signalling pathways are being identified, which ensure that physical activity stimulates AHN and improves memory function. In addition to BDNF, NGF, GDNF, EPO and VEGF, mentioned above, physical exercise was found to stimulate AHN through dihydrotestosterone [238], which, not surprisingly, mediates the well-known antidepressant effects of physical activity [239]. Direct support of a link between androgen activity and AD came from a recent study that provided evidence that androgen-deprivation as part of a prostate-cancer treatment doubled the risk of developing AD [240].

GH [241] and IGF-1 [242] also respond to repeated bouts of aerobic exercise, stimulate AHN and are neuroprotective. Fibroblast growth factor 2 (FGF-2) is also induced by physical exercise directly in the hippocampus [243] and was shown to restoring hippocampal function in murine models of AD [244]. FGF-2 is even discussed as a potential treatment option for AD [245], although, as the LOM predicts, single therapeutic measures have a low probability to be successful in curing AD. Furthermore, the central release of serotonin is required for physical activity-dependent AHN, where it also plays a direct and acute regulatory role in young adult as well as in aged mice [246]. This finding led to the conclusion that the understanding of exercise-induced AHN might offer preventive but also therapeutic opportunities in depression and age-related cognitive decline. Similarly, fat-cell secreted adiponectin appears to mediate physical exercise-induced AHN [247], thereby acting as an antidepressant [248]. Interestingly, some hormones or cytokines are secreted directly by the working muscle itself, which therefore can be considered as an endocrine organ [82]. The AHN-promoting hormones irisin [249] and meteorin-like [250] appear to be released solely by the active muscle.

Last but not least, the effects of physical activity on health and memory are also mediated by moderate levels of cortisol [251]. Whereas the positive effects of physical exercise peak at upper-middle intensity levels and thereby activating AHN, excessive cortisol levels due to extreme physical workout are inhibiting AHN [252]. One mechanism besides psychological stress that often accompanies extreme physical activity might be the increased release of pro-inflammatory cytokines like IL-6 from strained muscles that activate adrenocorticotropic hormone (ACTH) secretion from the pituitary gland, which, in turn, induces high levels of cortisol secretion [253]. This cortisol hypersecretion was found to aggravate neuroinflammation [254]. In contrast, regular and moderate physical exercise reduces systemic inflammation [255]. Similarly, while moderate physical activity enhances antioxidant defence mechanisms, intense levels of physical activity deplete the antioxidant reserve [256]. Therefore, extreme levels of physical exercise should be omitted as they are leading to adverse neurological effects [257]. Hence in order to maintain physical and mental health, a balanced life style with frequent workouts at moderate intensity is ideal, while physical inactivity (as well as over-activity) needs to be omitted whenever possible.

Besides the insufficient activation of AHN [258] that initiates the above outlined detrimental causal chain of events, physical inactivity directly increases the risk of developing AD by reducing the clearance of excess Aβ. A sedentary lifestyle was found to downregulate low density lipoprotein receptor-related protein 1 (LRP1), the major export protein for Aβ [259], located in the BBB. Physical inactivity also inhibits the activation of neprilysin, one of the main Aβ-degrading enzymes [260]. In addition, being sedentary leads to persistent sterile neuroinflammation [237], insulin-resistance, diabetes mellitus and visceral obesity. According to a recent study, these conditions all add to individual risk for developing AD [261].

Important for therapeutic considerations, becoming physically active even helps when AD is already diagnosed. A recent pilot study investigated the effect of a home-based physical activity intervention program on AD-patients on the development of clinical symptoms and functional abilities and the effects on family caregiver burden after 12 and 24 weeks [262]. While the (sedentary) control group experienced decreases in the ability of daily living performances, the patients in the (physically active) intervention group remained cognitively stable. Analyses of executive function and language ability revealed considerable positive effects for semantic word fluency in the intervention group. In fact, the physical active patients improved during the intervention, whereas the controls revealed continuous worsening. Consequently, caregiver burden remained stable in the intervention group but worsened in the control group.

AS the LOM tells us, physical exercise is not enough to effectively prevent or cure AD. Nevertheless, in most studies, the LOM was ignored and in some studies, in addition, the amount of daily exercise of the intervention group was very low, which led the researchers to devalue exercise for the prevention of AD [263]. Unfortunately, studies of this type, which are scientific standard (only one variable should be altered), lead to misleading results and their popularisation potentially undermine the public understanding regarding the importance of a healthy lifestyle. But being physically active is just one natural (and from an evolutionary perspective obvious) requirement that needs to be fulfilled. Another is the steady supply with essential nutrients.

### Nutritional effects on AHN

There is a long list of nutrients that are essential building blocks for (adult) neurogenesis and synaptogenesis (for review, see [264]) and which are also important for NRJ. For instance, n-3 polyunsaturated fatty acids (PUFAs), flavonoids, antioxidant-rich berries and resveratrol, a polyphenol found in red grapes and other fruits, were all shown to stimulate AHN [265]. In addition, they reduce oxidative stress and down-regulate pro-inflammatory processes (for review, see [266]); some even have potent anti-amyloidogenic properties [267, 268].

In contrast, a typical WeDi with its high intake of red and processed meats, animal fat, refined grains and sweets is low in essential nutrients and when simulated in animal studies, markedly reduces brain levels of neurotrophins such as BDNF, hampers neuronal plasticity and learning [269]. This type of modern diet also leads to increased ROS levels and neuroinflammation, which also hampers AHN and dysregulates the HPA axis by chronic cortisol hypersecretion [270]. There is also considerable evidence that advanced glycation end products (AGEs) might play a role in AD [271]. Sugar, fructose, corn syrup and generally food including beverages with high glycemic load cause intermittent hyperglycaemic episodes. This contributes to the endogenous generation of AGEs. In addition, meat products, particularly from industrial livestock farming, provide a large amount of exogenous AGEs (for review, see [272]). AGE (not age!)-induced neuroinflammatory processes are mediated by inflammatory cytokines like interleukin-1 (Il-1) [273], interleukin-6 (IL-6) [274] or tumour necrosis factor-alpha (TNF-α) [275] and aggravate neurodegenerative processes and were shown to further decrease productive AHN [276].

Intensifying the problem of chronic neuroinflammation caused by AGEs, is the high intake of omega-6 (n-6 PUFAs) in absolute, but also in relative quantities to n-3 PUFAs, whose intake is typically low in a typical WeDi [277]. In general, chemokines derived from the n-6 PUFA arachidonic acid (AA) are pro-inflammatory, while those derived from n-3 PUFAs, like for instance docosahexaenoic acid (DHA), have anti-inflammatory properties [278]. Pro- and anti-inflammatory processes are essential for wound repair and healing, respectively, but require a balanced intake of both types of PUFAs. But with the advance of industrially produced food, there has been a dramatic change from an healthy (and “natural”) n-6 PUFA to n-3 PUFA ratio of about 1 to 1 in pre-modern diet to ratios up to 20 to 1 in current WeDi [279]. One reason for this development is the widespread use of vegetable oils with high a high concentration of n-6 PUFAs, i.e. linoleic acid like e.g. sunflower oil (contains up to 70 %) and corn oil (up to 60 %). The use of these polyunsaturated oils was propagated by the food industry for their serum cholesterol-lowering properties, but recent study based on available evidence from randomized controlled trials shows that replacement of saturated fat in the diet with linoleic acid actually increased the risk of death from coronary heart disease or all causes by 22 % for each 30 mg/dL (0.78 mmol/L) reduction in serum cholesterol [280]. A healthy alternative would be the use of extra-virgin olive oil [281–283], as well as the moderate use of organically grown canola [284] or flaxseed oil [285], with healthier n-6 PUFA to n-3 PUFA ratios.

Another reason for the increase of n-6 PUFAs in our modern diet results from the intense consumption of meat and high fat milk products. The fact that those products are derived from intensive animal farming aggravates the negative effect on consumer’s health, since the unnatural “way of life” of those animals negatively impacts the n-6 PUFA to n-3 PUFA ratio in their products [286, 287]. Reason might be food for fattening, a lack of exercise and stress of the farmed animals. Last but not least, since the conversion of plant-derived n-3 PUFAs to DHA (or eicosapentaenoic acid (EPA), another physiologically important n-3 PUFA product, is a very inefficient metabolic process in humans, the low consumption of fish (the major source of animal DHA and EPA) in a WeDi leads to an absolute deficit in the supply of these two n-3 PUFAs. In addition, the high intake of n-6 PUFAs further compromises the already low metabolic conversion rate of plant-derived n-3 PUFAs to DHA and EPA [288]. The resulting serious imbalance of n-6 to n-3 PUFAs drives premature aging, neuroinflammatory processes, depression and AD [289, 290].

This development is unfortunate, since high concentrations of DHA would inhibit lipid peroxidation [291], and, together with EPA, would reduce brain inflammation and cognitive impairment [292]. Furthermore, many different neuroprotecting cellular mediators derive from DHA, EPA as well as another important n-3 PUFA, docosapentaenoic acid (DPA) (for review see [293]). For example, DHA-derived neuroprotectin D1 (NPD1) induces signalling for homeostatic maintenance of cellular integrity, and is neuroprotective due to its ability to inactivate pro-apoptotic and pro-inflammatory signalling pathways [294, 295]. Furthermore, NPD1 is a mediator with proven anti-amyloidogenic bioactivity [296], which was originally ascribed to DHA [297]. DHA was shown to positively regulate BDNF [298] and GDNF [299], thereby acting synergistically to enhance AHN and synaptic growth. In addition, PUFAs like AA and DHA are essential building blocks for all neuronal tissue, making up roughly 30 % of our brains membrane lipids [300]. Taken together, a diet deficient of DHA limits neurogenesis, making its supply critical for healthy brain development and maintenance of neural plasticity in later life [301]. Supporting these observations is the recent finding, that lower intakes of nutrient-dense foods and higher intakes of unhealthy foods, typical for a WeDi, were each found to independently being associated with smaller left hippocampal volume in humans [302]. In contrast, supplementation of about 1 g per day of a mixture with n-3 PUFAs DHA and EPA significantly improved episodic memory outcomes in older adults with mild memory complaints [303].

It is reasonable to assume that at least for the hundreds of thousand years of human evolution before the advent of the so-called “modern life style” beginning in the 19^th^ century, mankind was used to a diet with sufficient and roughly equal quantities of n-3 PUFAs to n-6 PUFAs (for review see [297]). It might be not only a mere coincidence that the change to a marine food diet which provided a stable and continuous supply of essential DHA (and EPA) sources appear to happen together with advancements in human’s cultural development, as exemplified by the bladelet stone tool technology and other cultural advancements [304]. Indeed, there is also now incontrovertible support from fossil evidence for the hypothesis that steady access to the marine food web must have played a critical role in human’s final steps in evolution [305]. This might have allowed humans to evolve big brains even without the ability to efficiently convert plant-derived n-3 PUFAs into DHA and EPA. Hence, humans are not at all adapted to the very recent and rapid change in dietary habits that cause a detrimental deficit in DHA as well as EPA.

While a lack of essential n-3 PUFA is causing neuroinflammation and a disturbed AHN (wherefore supplements containing EPA and DHA were shown to have a positive effect on primary depression [306]), there is also substantial convergence of research findings to date indicating that seafood consumption reduces the risk of dementia and AD [307].

However, not all researchers agree with this, since a major breakthrough in AD prevention by adding fish to patients’ diet has not been achieved (for a list of trials see [289]) and many therefore argue for longer-term human trials in hope for better success rates [308]. Notwithstanding, one should be aware that only those participants of any cohort might benefit from DHA treatment (or any other AHN-compromising deficit), who actually have a deficit in DHA (or any other respective deficit), and, according to the above outlined LOM, have no deficit in any other essential requirements for a productive AHN and NRJ. Hence, even if longer intervention trials might not raise the success rates, as long as we do not rectify all concomitant deficits. In any case, one should not conclude that providing sufficient DHA is not essential, simply because in most published cases it has on its own not been effective in preventing or treating AD (or major depression).

One key question however remains: Where should a steady world supply with DHA (and EPA) come from? With regards to meeting the assumed global consumption needs, there is a growing concern about the sustainability of global fisheries. An economic and ecological alternative to selectively consuming wild caught and organic farmed fish is to increase the supply of edible oil rich in DHA and EPA derived from of organically grown micro-algae [309]. This would also circumvent the problem of the contamination with methylmercury (MeHg) in fish (see below).

### Mediterranean diet (MeDi)

A healthy alternative to the standard WeDi is the MeDi [310]. Since all Mediterranean countries have their own type of cuisine, one has to be aware that what we call MeDi is an artificial construct. Nevertheless, the principal aspects of this diet include proportionally high consumption of olive oil, legumes, unrefined cereals, fruits and nuts, and vegetables, moderate to high consumption of fish, restrained consumption of dairy products, modest red wine consumption, and low consumption of non-fish meat and non-fish meat products [311]. Older adults, adhering to a MeDi, have less brain atrophy, with an effect similar to 5 years of aging, when compared to those adhering to a WeDi, according to a recent study [312]. Particularly higher fish and lower meat intake might be the two key food elements that contributed to the benefits of MeDi on brain structure, as the authors of the study pointed out. Another study provided evidence that such dietary interventions play a role in the prevention of AD. Here, individuals without cognitive deficits showing lower adherence to a MeDi had cortical thinning in the same brain regions as clinical AD patients, i.e. entorhinal cortex, orbito-frontal cortex, inferior parietal lobule, inferior and middle temporal cortex and posterior cingulate cortex, compared to those showing higher adherence [57]. Interestingly, ApoE4-subjects showing higher MeDi adherence had the greatest benefit of all other subgroups, which is in line with the aforementioned role of ApoE4 being primarily an accelerator of the AD process under unhealthy lifestyle conditions. A similar protective effect was found for the traditional Japanese diet (for review see [313]), which might explain at least partially the low AD prevalence rates in the old Japan, as discussed above.

Importantly, the benefits of the MeDi, like for any diet protecting against chronic disorders, should not only be attributed to its products. They are also strongly influenced by the food processing techniques and the style of cooking. Mechanistic and epidemiological evidence provide convincing evidence that food processing impacts on the quality of the inherent phytochemicals and this in turn impacts on the protective properties of these foods against chronic diseases associated with inflammation (for review see [272]).

### Vitamins

A WeDi is generally low in vital nutrients and leads to a reduced intake of vitamins and essential trace elements. In line with the LOM, each individual deficit inhibits productive AHN and was shown to increase the risk of AD, including deficits of vitamin A [314], all the B-complex vitamins [315], vitamin B1 [316, 317], B3 [318, 319], B6, B9 and B12 (see below), C (for a comprehensive review see [320]), D (see below and [321]), and the E tocopherols [322]. As it is beyond the scope of this review to detail the impact of each deficit on AHN and resulting risk for depression and AD, I will provide only two examples. Nevertheless, in AD prevention (and therapy), each deficit needs to be addressed.

#### Example 1

Nutritional deficiencies in the either of the vitamins B6, B9 or B12 lead to an increased accumulation of homocysteine, an intermediate product in the L-methionine and L-cysteine amino acid metabolism, where these three vitamins are essential cofactors. An elevated homocysteine blood level is a predictor of cognitive decline in AD [323]. Homocysteine induces oxidative stress through neuronal nitric oxide synthase activation and free radical formation [324], thus causing a significant increase in neuronal cell death [325]. Furthermore, elevated homocysteine was shown to be detrimental to AHN [326], where it inhibits the proliferation of neuronal precursors by interfering with several signalling pathways required for cell proliferation [327]. A recent 10-year study based on post-mortem neuropathological and MRI findings identified that elevated homocysteine was related to Aβ accumulation and brain atrophy. The highest homocysteine quartile had an odds ratio of 3.78 for medial temporal atrophy and 4.69 for periventricular white matter hyperintensities [328]. Since all associations were independent of several potential confounders, including common vascular risk factors, deficiencies of either vitamin B6, B9 or B12 should therefore be regarded as independent risk factors for AD due to homocysteine elevation.

However, despite the fact that lowering homocysteine-levels by supplementing B vitamins was shown to significantly slow-down the rate of accelerated brain atrophy in MCI (from 1.08 % per year in the placebo group to 0.76 % in the active treatment group) [329], no full prevention nor cure of AD can be expected, according to the LOM, as long as other essential risk factors are not eliminated as well. Only a preventive and therapeutic scheme that provides all essentials, as schematically outlined in Fig. 1, might be successful. Therefore, management of potential micronutrients deficiencies can only be one, albeit important, aspect of AD prevention and treatment strategies (for review see [313, 330]).

#### Example 2

Vitamin D deficiency is known to decrease bone density and increase the risk for many common forms of cancer [331] as well as cognitive impairment in older but also younger adults [332]. The overall prevalence rate of vitamin D deficiency is widespread. For instance, it averages in the USA at about 41.6 %, with the highest rate seen in blacks (82.1 %), followed by Hispanics (69.2 %), according to a recent study [333]. Vitamin D deficiency causes abnormal AHN [334]. While the results of a recent meta-analysis analyses were consistent with the hypothesis that low vitamin D concentration is associated with depression (taken as a sentinel disease for an unproductive AHN), there is always the call for more randomised controlled trials to find out for instance, if vitamin D supplementation could indeed be useful for the prevention and treatment of depression [335] or cognitive decline [336]. I understand that there is a strong scientific need to understand better how Vitamin D insufficiency dysregulates certain nerve growth factors and respective signalling pathways [337], but waiting for more scientific understanding what goes wrong when an essential vitamin is missing does not help those who suffer right now under such a deficiency and are prone to develop serious diseases. Furthermore and again, such interventions do not help those included in trials that have sufficient serum vitamin D at baseline [338]. Second and as stated above, only those participants benefit fully of vitamin D supplementation when at the same time all other essential factors, that are missing and which are required for a productive AHN are provided as well. Since this is rarely done, a weak or negative outcome regarding AD prevention should not lead to the false conclusion that the deficit in question is not casually linked to AD and should be corrected.

Indeed, the results from a recent study confirm that vitamin D deficiency is strongly associated with a substantially increased risk of all-cause dementia and AD [339]. Conversely, higher dietary intake of vitamin D was shown to be associated with a significant lower risk of AD [340]. Furthermore, a recent study provided evidence that serum 25-Hydroxyvitamin D levels of 70 nmol/L were associated with the lowest cardiovascular disease mortality risk [341], close to the values that showed an over two-fold reduced brain atrophy (>50 nmol/L), when compared to insufficient vitamin D levels [339]. In my opinion, all the results taken together warrants immediate action of primary care physicians but also policy makers to make the screening for deficits in all essential nutrients, Vitamin D being one of them, part of nationwide prevention programs.

### Trace elements

The same logic in the prevention and treatment of AD applies to deficiencies in essential trace elements, like for example selenium, zinc or lithium.

*Selenium* deficiency plays an important role in AD [342, 343], because selenium-dependent enzymes (selenoenzymes) are required to prevent and even reverse oxidative damage throughout the body, and in particular in the nervous system [344]. An optimal range of serum selenium around 85 μg/L was associated with reduced risk of depressive symptomatology in young adults [345]. Interestingly, inducing differentiation in primary neuronal stem cells (NSCs) was recently shown to result in an immediate increase of total mitochondria number and overall ROS production, suggesting oxidative stress is generated as a result of routine adult neurogenesis [346]. This metabolic adaptation process appears to be common for all types of stem cell systems [347]. Therefore, a deficiency of selenium seems to limit the buffer capacity against an AHN-increased ROS production, which might result in an non-productive AHN and therefore a heightened risk of depression and AD.

*Zinc* is another main trace element required for AHN [348, 349]. In patients with major depression, a low zinc serum level was correlated with an increase in the activation of markers of the immune system, leading to impaired AHN. Moreover, a preliminary clinical study demonstrated the benefit of zinc supplementation in antidepressant therapy. In preclinical research, the antidepressant activity of zinc was observed in the majority of rodent models of depression and revealed a causative role for zinc deficiency in the induction of depressive-like symptoms, the reduction of neurogenesis and neuronal survival or impaired learning and memory ability (for review see [350]). In addition, zinc is critical for the enzymatic non-amyloidogenic processing pathway of the amyloid precursor protein (APP) and for enzymatic degradation of the Aβ [351].

*Lithium* is not yet generally recognized as a trace element but several lines of evidence make it a strong candidate [352]. For instance, long-term low-dose exposure to lithium exerts anti-aging capabilities and unambiguously decreases mortality in animal models [353]. In humans, epidemiological studies indicate an inverse correlation between lithium concentration in drinking water and mood, depression and suicide rates [354], amongst other psychiatric conditions [355]. In a study that compared elderly bipolar patients (who exhibit a higher risk for dementia) who had received chronic lithium treatment, with bipolar patients who had not received lithium, it was shown that the prevalence of the treated group was equivalent to the general, age-comparable population, whereas the non-lithium-treated patients had an incidence of dementia that was six times greater, i.e. 5 % vs. 33 %, respectively [356]. In another study it was shown that lithium treatment resulted in an increase in volumes of the hippocampi in both hemispheres compared to an unmedicated group, an effect that was apparent even after a brief treatment period of about 4 weeks on average [357]. Importantly, intake of lithium not only in standard therapeutic but also in trace doses reduces the risk for dementia, suicide, and other behavioural outcomes, suggesting an pharmacological interference with key regulators of these pathological processes [358]. So, lithium naturally regulates critical cell signalling pathways and a lack of lithium in the diet can therefore cause increased disease risk.

It has been shown that lithium modulates negatively the activity of the two kinases GSK-3α and GSK-3β, which might explain both the relative specificity and sensitivity of the effects of low-dose lithium treatment (see below) [359]. Since GSK-3β-activation by oligomeric Aβ promotes neuroinflammation, phosphorylation of tau and disturbance of AHN [360], all key mechanisms in the AD process, in inhibition of GSK-3 by lithium results in reduced tauopathy and neurodegeneration in vivo [361]. Likewise, lithium treatment was shown to improve AHN, neuropathology and cognitive functions in a mouse model of AD [362]. Furthermore, such “AD-mice” treated from two months of age had decreased numbers of senile plaques, no neuronal loss in cortex and hippocampus and increased BDNF levels when compared to non-treated transgenic mice [363]. In order to achieve this effect, it was sufficient to give lithium at about one per mill of the high standard-dose therapy in bipolar disorder, a dose which can cause some significant side effects. Hence the authors of the study believe that their data support the use of (virtually side-effect free) microdose lithium in the prevention and treatment of Alzheimer’s disease.

Indeed, long-term lithium treatment already provided preliminary evidence of its disease-modifying properties for amnestic MCI in a randomised controlled trial, where the lithium-treated group had fewer conversions from MCI to AD. [364]. Lithium treatment was associated with a significant decrease in cerebrospinal fluid concentrations of hyperphosphorylated tau and better performance on the cognitive subscale of the Alzheimer’s Disease Assessment Scale and in attention tasks. At a more advanced stage of AD, microdose treatment with only 300 μg lithium administered once daily stabilized the AD patients during the complete evaluation phase of 15 months [365]. For instance, whereas the treated group showed no decreased performance in the mini-mental state examination (MMSE), lower scores were observed for the control group during the same period, with significant differences occurring after three months, and increasing progressively.

Importantly, lithium, besides being required for efficient AHN and blocking AD-specific pathological processes, also impacts on cell-rejuvenating autophagy. Lithium was found to inhibit the activity of inositol monophosphatase (IMPase), which leads to a decrease of myo-1, 4, 5-triphosphate (IP3). This reduction of IP3-activity induces autophagy, independent of mTOR [366]. In this context it is important to note that lithium chloride extends the lifespan of the nematode Caenorhabditis elegans [367], possibly by means of mitochondrial rejuvenation [368], which suggests that lithium exerts its effects on evolutionary highly conserved mechanisms. Intake of drinking water with comparable low lithium concentrations were found to be inversely correlated with all-cause mortality in a large epidemiological study in Japan [353]. Hence a lack of lithium is linking aging and frailty (all increasing mortality) to disturbed autophagy, and AD to impaired AHN, and, thus, might represent another important and modifiable risk factor in this neurodegenerative disease [369]. Traces of lithium can be ingested in some geographic areas by drinking local tap water or otherwise by consuming commercially available, mineral-rich spring waters, containing suitable concentrations of around 1 mg lithium per litre. Hence microdose lithium intake by means of one or two glasses of such water a day is not only of potential therapeutic value (see below) but also a safe preventive measure, by means of simply reducing an intake-deficit of an important novel trace element.

### The importance of IMF for brain fuelling, AHN and neuronal rejuvenation

Hunting and gathering are perilous enterprises. Hence leaving the secure living area and going into the wild was most certainly avoided by our ancestors as long as their stomach was filled. As a consequence, mechanisms must have evolved that allowed brains and bodies to functioning well even when hungry. Particularly the episodic memory function of the hippocampus is important, since remembering well where food can be found and predators are lurking was key to survival and therefore reproductive success. Linking IMF to hippocampal growth was such a mechanism our ancestors could rely on (see below). But whereas the need to acquire food was a major day-to-day challenge during much of hominid evolutionary history, for people in modern societies a constant supply of food became the norm [66]. The resulting ad libitum eating pattern also applies routinely to laboratory animals. For example, transgenic AD models kept in standard housing with ad libitum feeding develop AD-like symptoms, and a high high-caloric diet even accelerates memory impairment [370]. In contrast, keeping such mice under a more natural IMF-diet improved cognitive functions and brain structures not only in wild-type mice [371]. One year of IMF prevented cognitive decline even in a triple-transgenic mouse model of AD, which expresses the human APPswe, PS1M146V, and tauP301L mutations [42], providing evidence that not aging per se but rather long-term feeding patterns have long-lasting effects on memory [43]. Interestingly, the positive effects of IMF were independent of changes in Aβ and tau pathology, hinting that IMF protects brain cells by another mechanism, like for instance by activation of protein chaperones, antioxidant enzymes and by the suppression of neuroinflammation [372–374] or by activating NRJ [375], thereby promoting regeneration, enhanced cognitive performance, and healthspan [376]. Furthermore, the “hunger hormone” acyl-ghrelin was found to activate proneurogenic signalling pathways by increasing levels of the neurogenic transcription factor Egr-1 in DG-neurons [377]. IMF also induces FGF2 and BDNF expression in the hippocampus [378], which promotes the survival of newly generated neurons and thereby contributes to the enhancement of AHN [379]. BDNF also protects DG-neurons against excitotoxicity, energy deprivation and oxidative stress [380, 381].

Another hallmark of AD is increased neuronal insulin resistance in the temporal lobe. The inability of insulin-resistant hippocampal neuronal cells to take up glucose is another driving force of the AD process [382]. Due to “standard housing” in animal research and the “normality” of the Western lifestyle as observed in epidemiological studies, the detrimental condition of neuronal insulin resistance is similar to AD regarded as a primary consequence of aging, and rarely as result of a sedentary lifestyle, obesity, a WeDi, high cortisol secretion and the accumulation of toxic Aβ-levels caused by increased production (impaired AHN) and disturbed removal. Conversely, an experimental change to an IMF-like dietary pattern as well as to more daily physical activity increases insulin sensitivity, fatty acid mobilization and ketone body production (which circumvents the energy starvation of hippocampal neurons in AD). Importantly, these lifestyle adaptations, which take advantage of our genetic program, are not only important for AD prevention but should also be an indispensable part of any prevention and curative treatment program for many chronic disorders [383].

Intriguingly, the ketone body βOHB is not only a carrier of energy from the liver to peripheral tissues like the brain during IMF or exercise, βOHB was also found to be an important signalling metabolite [91]. For example, βOHB acts through the adipocyte receptor HCAR2 (hydroxycarboxylic acid receptor 2), also known as GPR109A, through which niacin (Vitamin B3) stimulates the secretion of adiponectin [318], known to induce AHN [247]. By activating HCAR2, βOHB also negatively regulates its own production, thereby preventing ketoacidosis and promoting efficient use of fat stores. In addition, βOHB suppresses the sympathetic nervous system (which leads to energy conservation when fasting) by antagonizing FFAR3 (free fatty acid receptor 3), also known as GPR41, which is most abundantly expressed in sympathetic ganglia of mice and humans [384].

Furthermore, by inhibiting histone deacetylases (HDAC), βOHB regulates via epigenetic reprogramming the expression of genes that support subcellular renewal and cellular survival, like for instance autophagy and stress response pathways, which leads to neuroprotection and longevity. These genetic programs are highly conserved. For instance, βOHB, by inhibiting HDACs, extends the lifespan of C. elegans by a multitude of mechanisms including the activation of conserved stress response pathways, increased thermotolerance, reduced glucose toxicity and, in regard to AD, delayed toxicity of oligomeric Aβ [385]. Similarly, βOHB protects hippocampal cultures from hypoxia and glutamate excitotoxicity [386], as well as Aβ-toxicity (for review see [387, 388]). In addition, βOHB counteracts neuroinflammation [389], protects against insulin glycation (insulin-AGEs) as a mechanism for neuronal insulin resistance, and increases microglia viability by an anti-lipid peroxidation effect [390]. βOHB also stimulates autophagy and prevents neurodegeneration induced by glucose deprivation in cultured cortical neurons [391]. Furthermore, βOHB-induced upregulation of hypoxia inducible factor-1α, a primary constituent associated with hypoxia-induced angiogenesis and a regulator of neuroprotective responses, leads to a reduction in neuronal loss in stroke models [392]. And, as outlined above, increasing ketogenesis by extensive physical activity or IMF leads to activation of PGC-1α and inhibition of mTOR, and consequently longevity, at least on the neuronal level.

In this context it is important to note that the health benefits of IMF do not require overall reduction in caloric intake, like for instance CCR. For example, mice fed only for 8 h a day (i.e. IMF for 16 h daily) are resistant to high-fat diet-induced obesity [393], without altering the overall calorie intake. The longer the ketosis on a day to day basis the better for health outcome, which even puts into question the widely held belief that breakfast is the most important meal of the day [394]. Indeed, it is reasonable to suppose that skipping breakfast could be as metabolically beneficial as excluding late eating, as long as an inter-meal interval is long enough to allow sufficient ketogenesis to reduce the risk of obesity and of co-morbidities like AD [395].

Taken together, the endogenous signalling metabolite βOHB improves memory function not only by providing brain fuel under conditions of neuronal insulin resistance, but also by favourably changing our genetic and epigenetic program. This is an indication that the principles of long-term brain health as they developed during evolution still are valid today [396]. Besides IMF and prolonged physical activity, brain-protecting ketogenesis remains active during the day if we omit the intake of food with a high glycemic index that leads to a postprandial secretion of insulin, as this “sugar-stress” hormone most effacingly blocks lipolyses [397] and ketogenesis [398]. Furthermore, there even exists a natural possibility to increase ketogenesis when eating.

### Ketogenic brain fuelling without fasting

The ingestion of medium-chain triglycerides (MCTGs), like those being provided by virgin coconut oil, which contains them at concentrations up to 60 %, leads to elevated ketone body production. In contrast to ingested long-chain triglycerides, MCTGs are water soluble and therefore directly transported via the portal vein to the liver where they are efficiently transformed into ketone bodies [399]. And indeed, the acute administration of MCTGs appears to improve somewhat memory performance in AD patients [400, 401], whereby the degree of memory improvement was found to being positively correlated with plasma levels of generated βOHB. But the benefits were only observed in less advanced stages of the disease [402]. Furthermore, in all these studies, besides the weakness of the overall beneficiary effect, they were less pronounced in ApoE4-carriers. Both these observations might indicate that using MCTGs like a drug to increase βOHB-levels does not completely provide all possible health benefits generated by IMF or prolonged physical activity. Hence as important as ketogenesis for hippocampal metabolism, AHN and neuronal rejuvenation might be, simply adding ketogenic MCTG to the diet is not sufficient to prevent or treat AD. Nevertheless, some researchers tend to base a complete unified theory of AD simply on activation of ketogenic energy metabolism [403].

Comparisons of the effects of energy restriction and physical exercise on gene expression in various brain regions support the notion that these two different environmental factors have both overlapping but also distinct effects on brain cell plasticity [66]: While running exercise primarily enhances the proliferation of neural stem cells and secondarily the survival of newly generated neurons, IMF, besides supporting basal stem cell proliferation, first and foremost improves the chance of survival of newly generated neurons [379]. In this regard it is obvious that exercise and energy restriction show additive effects on AHN [404] as well as neuroplasticity: Both running wheel exercise and daily energy restriction resulted in increased dendritic spine density in hippocampal DG neurons when compared to mice that only run or are only energy restricted [405]. From the evolutionary perspective, these findings are consistent with the notion that IMF and foraging result in structural and functional improvements of neuronal circuits in the brain that provide a survival advantage. These physiological effects explain why human intervention studies fail to show clear cut effects when they focus on only one aspect (like only providing only coconut oil or MCTG or advocating only IMF as a lifestyle choice). It also explains, why a WeDi, where most individuals on a daily basis consume large amounts of simple sugars and unhealthy fats in large quantities become are chronically ill. Under these conditions, a large number of people are not only deprived of food with high nutrient value, also efficient and therefore health-protecting ketogenesis rarely occurs [406, 407].

Taken together, besides the importance of IMF and physical exercise, also dietary MCTGs can be an attractive (indirect) source of ketone bodies during the non-fasting state. The intake of coconut oil as a healthy source of MCTGs has additional positive effects on neuronal insulin resistance, neuroinflammation as well as Aβ-toxicity, improves the LDL/HDL-cholesterol quotient by increasing HDL, and ameliorates several others key progression factors of AD [408]. Hence the use of virgin coconut oil is highly recommended as a safe and healthy alternative for polyunsaturated oils for frying and baking and butter and an important part of a comprehensive strategy for the prevention and treatment of AD.

### Body composition and visceral fat

A lifestyle that encompasses 12+ hours IMF overnight, a nutrient-rich, low-glycemic diet and regular physical exercise almost inevitably leads to a body composition close to that of our foraging ancestors: lean and muscular. Unfortunately, the behavioural trend goes in the other direction, where obesity and “age-related” muscle loss is becoming a major health issue [409]. This trend has negative consequences also on brain function, since adipocytes produce adipokines (like muscle cells myokines) with endocrine functions that affect the physiology of essentially all organs, including the brain [410]. Both, the lack as well as the abundance of visceral fat leads to an unhealthy regulation of these adipokines. In particular, the epidemic visceral obesity causes a rise of several disease risks for AD like the development of chronic inflammation [411], insulin resistance [412], hypertension [413], hypercholesterolemia [414], arteriosclerosis [415] and finally, the metabolic syndrome, a condition composed of more or less all these AD-risk factors cited above [416–418].

The dysregulation of certain adipokines have also direct effects on the proposed central mechanism of AD, namely AHN. For instance, leptin is an adipokine well-known for its hypothalamic regulation of food intake and energy metabolism. But leptin has also remarkable effects on AHN, axon growth, synaptogenesis, dendritic morphology, neuron excitability, neuroprotection and even regulation of Aβ levels [419]. Due to these pleiotropic effects, leptin treatment was shown to reduce brain pathology and to improve memory in transgenic mouse models of AD [420]. Conversely, obesity induced overexpression of leptin causes leptin resistance and hence disturbed AHN and depression [421]. In a murine model for obesity and diabetes, leptin resistance was shown to promote AD-relevant tau pathology [422]. Conversely, the neuroprotective adiponectin, a prominent activator of AHN, is down-regulated by central obesity [423].

Taken together, adipokines impact directly and indirectly on key brain functions, which adds central obesity in midlife to the large list of causal risk factors for AD [extensively reviewed in [424] and [425]. Hence any causal AD prevention strategy should aim to reduce visceral fat depots by encouraging physical activity, overnight IMF of at least 12 h, and a healthy diet (e.g. MeDi).

### Social engagement and purpose in life

Brains have for one central function: to overcome environmental challenges in order to improve the chances of survival and successful reproduction. AHN in particular is important as it allows the lifelong collection of essential life experiences. But cognitive capabilities are only improved by cognitive challenges. Without new experiences, newborn DG-neurons would not interconnect with the existing DG-network. Without functional synapses, neurons undergo apoptosis [426]. Hence for a productive AHN to occur, the human brain continues to require memorable activities even at high age. Since social activities for beings like humans, whose survival depend on social support, have high importance and therefore a high emotional impact, they also generate best memorable experience. In other words, mental health is depending on social interactions. Neither retirement at old age was part of human’s long life history nor the modern individualism that leads ever more frequently to a breakdown of tight extended family bonds where grandparents are less (or not) involved in family support. The impoverishment in mental challenges and social interactions has detrimental consequences, since those individuals rating at a low score on the purpose in life measure were found to be associated with a 2.4 times increased risk of AD when compared to those rating themselves at a high score [427].

Since correlation in human studies does not a priori equal causality and sometimes even reflects reverse causality, animal models are important for distinguishing between different causal possibilities and in case of a causal correlation help to even possibly identify the underlying molecular mechanism. In animal research, experience of a complex challenging environment was shown to enhance brain plasticity, improving both structure and function. The effects of environmental enrichment (EE) are of particular importance under neuropathological conditions: Experience in a complex (natural) environment rescues impaired AHN in transgenic mice harbouring a familial AD-linked transgene [428]. Productive proliferation of hippocampal cells improved significantly after EE. In addition, EE significantly enhanced hippocampal LTP. Furthermore, enhanced AHN was accompanied by a significant reduction in levels of p-tau and oligomeric Aβ in the hippocampus and cortex of environmentally enriched mice. In another animal model of AD, social interaction rescued memory deficits by means of increasing BDNF-dependent AHN [429]. Prolonged enrichment was even found to prevent LTP inhibition by synaptotoxic Aβ-oligomers [153]. From these experiments one can conclude that environmental modulation might be able to rescue the impaired phenotype of the AD brain and that induction of brain plasticity by increasing social and cognitive challenges (and not decreasing, as often happens to patients diagnosed with early AD) may represent a valuable therapeutic and preventive avenue. Indeed, human studies revealed that higher baseline social integration predicted slower memory decline in a representative US sample of elderly adults [430]. Memory among the least integrated patients declined at twice the rate when compared with the most integrated ones. Additionally, there was no evidence of reverse causation in these study groups, i.e. cognitive decline causing reduced social interaction.

On the other hand, while systematic cognitive training and respective computer-based programs produce long-term improvement in cognitive function and less difficulty in performing activities of daily living, activities like these do not simulate social (emotional) learning and therefore might not as well improve AHN. i.e. the survival of newborn DG-neurons. Although computer-based mental training has it benefits for the skills being trained [431], it is therefore no surprise that cognitive training was not found to be associated with a lower rate of incidence in dementia [432]. From an evolutionary perspective, human’s ability to stay mentally healthy up to high age, based on a productive AHN, requires other activities. In particular and in line with the GMH, being and feeling generative by exhibiting concern and behaviour to benefit others (or at least kinship) is a very important developmental goal of midlife and beyond. Therefore, a recent study examined whether participation in the intergenerational civic engagement program, named Experience Corps (EC), would benefit older adults’ self-perceptions of generativity (as outlined above, generativity is meant in the psychosocial sense and refers to the concern for establishing and guiding the next generation). Participants randomized to the intervention group were placed as volunteers within the Baltimore public school system for 2 years. The results provided the first-ever, large-scale experimental demonstration that participation in an intergenerational civic engagement program not only positively alters self-perceptions of generativity in older adulthood [433], but also buffered against brain atrophy. In particular, whereas men in the control arm of the EC-program exhibited continued hippocampal declines for 2 years, men in the socially active arm showed a 0.7 to 1.6 % increase in brain volumes [434]. These findings show that purposeful social activity not only stops but even reverses the decline in brain volume in regions vulnerable to dementia, commonly observed in the elderly population.

In contrast, unintended by the individual or even forced social isolation is stressful and disrupts AHN, as was recently shown in marmoset monkeys [435]. Moreover, traumatic events early in life result in HPA axis dysregulation with deleterious consequences like anxiety-like behaviours later in life, which in turn continue to disturb AHN. In animal models, sensoring of circulating corticosteroid levels was shown to be impeded through epigenetic downregulation of the corticosteroid receptor (CR) in hippocampal neurons, potentially leading to long-term HPA axis dysregulation [436]. There is evidence that the same principles work in humans: DNA methylation levels in the promoter region of the GR gene were shown to be positively correlated in a cumulative manner with the number of stressful life events as child or young adult experiences (such as parental divorce, severe illness etc.) [437]. Reduced cortisol sensing hinders HPA axis negative feedback regulation, thereby increasing the risk to develop major depression or AD. For example, childhood abuse altered HPA stress responses and increased risk of suicide, which was found to be closely linked to epigenetic differences in a neuron-specific GR promoter [438]. Correspondingly, a chronically distressful life, like chronic problems at work or a chronic disabling disease, divorce, or death of a close family member, was shown to be inversely correlated with hippocampal size, which was linked to cognitive deficits in depressed patients [439]. According to a recent 38-year longitudinal population study, such psychosocial stressors in middle-aged women appear to increase the risk of AD in later life [440]. But not everybody who experiences stressful events becomes depressed or develops AD. Indeed, those who are psychological resilient and cope with such distressful events, as a more detailed follow-up analyses of the study data revealed, are less prone of developing AD later in life. The researchers found that a higher degree of “neuroticism” (characterized by anxiety, fear, moodiness, worry, envy, frustration, jealousy, and loneliness) in midlife was associated with long-standing distress and increased risk of AD dementia. In particular, personalities with high “neuroticism” and low extraversion scores showed the highest risk of AD dementia [441]. Both traits, “neuroticism” and introversion, are associated with HPA axis dysregulation [442, 443]. The authors of the population study conclude that midlife “neuroticism” is associated with increased risk of AD dementia, and that distress mediates this association. Hence not stressful events but our reaction to those stressors affect our mental health in the long run. The results of these studies have therefore implications for AD prevention as well as treatment of early AD: In both cases, increasing psychological resilience by reactivating a productive AHN must be a primary goal.

The required lifestyle changes reduce the risk of cognitive decline, but are generally regarded as stressful, at least in the beginning. But they also lead to rewarding experiences (more physical and mental fitness, more social activities) and thereby buffer progenitor cells in the DG from the negative effects of elevated stress hormones [254]. Particularly oxytocin is a potent activator of AHN even under stressful situations marked by high cortisol levels [444], and was found to positively modulate the behavioural and physiological responses to stressors [445]. In humans, oxytocin is released in the hippocampus under conditions of social reward [446]. Besides oxytocin, in particular the activity of neuropeptide Y (NPY) in the neurogenic “niche” of the hippocampus has been associated with behavioural resilience to stressors [447], mediating the positive effects of certain stressors/rewarding experiences on AHN [448]. Interestingly, the NPY-ergic system was recently found to be a key mediator of the EE-facilitated coping-with-stress response and reduced anxiety in an animal model for posttraumatic stress [449]. Since stress coping stimulates AHN in adult non human primates [450], psychotherapies designed to promote stress coping combined with AHN-promoting behaviours, as outlined in this review, should have similar beneficiary effects in humans [451]. In particular those subjects should profit from such an intervention, who belong to high risk groups for developing major depression or AD. As a consequence, yoga or mindfulness-based stress-reduction programs [452], as well as psychotherapy to overcome stressful life experiences of the past, thereby increasing psychological resilience, should be part of any AD prevention and treatment strategy [453].

### The importance of sleep

Sleep deprivation harms hippocampal learning and memory. As outlined above, AHN is particularly sensitive to the consequences of sleep loss [454]. In particular, hippocampal subfield atrophy is observed in chronic insomnia and sleep fragmentation leads to reduced AHN as well as neuronal loss in the cornu ammonis (CA) subfields, which renders patients with chronic sleep disturbance particularly vulnerable to cognitive impairment [455].

In addition to the manifold mechanisms by which sleep disruption causes an increase in AD risk [456], a lack of uninterrupted sleep disturbs melatonin’s function on brain health. Melatonin is secreted at the onset of darkness from the pineal gland to orchestrate deep sleep. Melatonin was found to be a potent inhibitor of neuroinflammation [457]. In addition, it promotes dendritic maturation of newly generated DG-neurons and supports their survival [458]. This proneurogenic hormonal effect is supported by hormonal mediators like GH and BDNF, which are being upregulated during sleep. Furthermore and as importantly for a productive AHN, during deep sleep, secretion of cortisol as well as the expression of antineurogenic immune mediators like IL-1, IL-6 and TNFα are being reduced. Importantly, Aβ concentrations, which rise during wakefulness, fall during sleep. If this Aβ diurnal pattern is compromised by inadequate sleep, accumulation and oligomerization of toxic Aβ occur [459]. Interestingly, Aβ deposition even in the preclinical stage of AD causes disturbed sleep quality [460], leading to a vicious circle that needs to be addressed in any systemic therapeutic approach.

Recently, the breakdown of the BBB was suggested to be a consequence of aging per se (similarly to neuroinflammation, type-2-diabetes or AD). The cause was found to be the accumulation of oligomeric Aβ in the hippocampus [11, 461]. Hence BBB dysfunction was seen as a cause and consequence of AD [462]. But as discussed, under natural condition, particular with sufficient REM sleep, our brain is protected from these putative effects of aging. Indeed, sleep restriction in general [463] and REM sleep restriction in particular was found to increase BBB permeability, but even brief periods of deep sleep rapidly and effectively restored even severe alterations of BBB function [464]. These observation suggests that REM sleep not only regulates the physical barrier properties of the BBB, but also that neither Aβ-accumulation nor BBB-permeability might not be an inevitable consequence of aging.

In this context it is noteworthy that sleep disruption is also the most common consequence of traumatic brain injury, which might explain the increased AD risk following concussions of professional NF-players (as discussed above). When frequently interrupted sleep becomes a chronic condition, no matter what the aetiology, it causes decreased cellular repair and neuroinflammation, compromised BBB function, altered neuroplasticity, neurodegeneration, a reduction of AHN and hippocampal volume and impaired hippocampal plasticity and function [142, 465]. Hence sleep inadequacy with increased daytime sleepiness are both risk factors for dementia, independent of demographic and clinical factors [466].

Taken together, improving sleep quality must be an important aspect in any regimen aiming to prevent or treat cognitive decline. Potential treatment options for restoring the normal sleep cycle are improving sleep hygiene (www.ncbi.nlm.nih.gov/pubmedhealth/PMH0072504), mindfulness training [467] and cognitive behavioural therapy, in particular when psycho-traumatic experiences need to be overcome. Medications like melatonin [468], analogues [469], or its precursor tryptophan (which is also a precursor of serotonin and a provitamin of vitamin B3) might help in acute situations until behavioural causes have been identified and removed.

### The complex mechanisms of lifelong brain health

Predicted from the GMH, human reproduction strategy (and genetic selection) relies on the lifelong acquisition of experience that increases the chance of survival of the following generations. As depicted in Fig. 1 and from an evolutionary view as outlined in the preceding text, human’s genetic program appears to be exquisitely adapted to a pre-modern lifestyle and optimized to provide the key requisite for transgenerational generativity of the experienced elderly: i.e. mental health up to high age. The daily supply with vitamins, essential PUFAs and trace elements, sufficient physical activity, IMF, a rich social life with a purpose up to high age as well as enough time for regeneration (sleep) most likely characterized environmental and behavioural “normality” for the largest part of human evolution. There was for instance no need for our ancestors to maintain genes that encode an energy-costly vitamin C-producing machinery, since sufficient vitamin C was always in the diet. One important physiological function that depends on the constant availability of all these disparate factors is AHN. But according to the LOM, AHN, like any biological growth process, can only be productive if all essential requirements are fulfilled. Adult-born DG-neurons regulate HPA axis activity by registering novelty and comparing it with prior experiences. A productive AHN might therefore lead to increased psychological resilience with an enhanced interest in new challenges (novelty seeking). This increases the likelihood of novel experiences, a prerequisite for neuronal maturation and long-term brain health (for review see [470]). I term this natural process, as indicated in Fig. 1, the “virtuous circle of mental growth”. Most of the behavioural and environmental factors that are required for a productive AHN also influence other important physiological functions of our organism like for instance insulin sensitivity, blood pressure and microcirculation, immune and BBB function or cellular rejuvenation, and thereby indirectly influence neuronal survival up to high age. All these behavioural factors optimize Aβ homeostasis, with Aβ-production during wake, assuring synapto- and neuroprotective concentrations, followed by Aβ-degradation during sleep in order to efficiently acquire new experiences by hippocampal learning. Taken together, under the guidance of our genetic program, all these complex environmental, social, behavioural, physiological and molecular processes appear to be needed to sustain lifelong mental health.

### AD is driven by deficiencies and accelerated by environmental toxins

In contrast and as shown in Fig. 2 (which contains all major risk factors and deficiencies leading to AD), and depending on each individual’s scheme of life, one or several qualitative deficits combine in different quantities and limit the efficient generation, maturation and/or of integration of new DG-neurons by AHN. As outlined above and depicted in Fig. 2, an ineffective AHN limits memory capacity and starts the “vicious circle of depression”. The proposed model reconciles the three conflicting models originally suggested by Sapolsky to explain the physiological link between hippocampal shrinking and depression [471], and it provides a logical basis for the many different causes of depression besides chronic or severe psychosocial stress. Direct causes are the aforementioned deficiencies in essential requirements for productive AHN (behavioural deficits) and the interference with a productive AHN by environmental toxins, which will be described below. Indirect causes are the behavioural consequences of those deficiencies (or toxins) that either disturb BBB-function, lead to neuronal insulin resistance, obesity, hypertension, arteriosclerosis and reduced blood supply and aggravate the behavioural deficits. None of them might therefore be caused by aging per se since unhealthy lifestyle conditions might suffice. Nevertheless, particularly neuroinflammatory processes, which inhibit AHN and lead to increased Aβ accumulation, are almost always regarded as a consequence of aging [472]. But is neuroinflammation indeed caused by aging per se, as often argued (for instance see [473]), or is it rather a consequence of modifiable lifestyle factors (and a misinterpretation of animal research under unnatural housing conditions)? Results of a recent study suggest the latter, as neuroinflammation was found to be far less pronounced in older AD patients when compared with younger AD patients [474]. If chronic inflammation as the driving force of AD was indeed age-induced, the opposite result should be expected. Neuroinflammation is rather multi-causal, with the same behavioural deficits and environmental factors contributing, which were found to disturb productive AHN (for review see [475]). In addition to the nutritional factors outlined above, these modifiable factors range from chronic stress [476], an amyloid-producing microbiome that might act as seeds for Aβ-oligomerization in the brain (for review see [477] as a consequence of inadequate nutrition, a sedentary lifestyle [478], poor sleep quality [479], visceral obesity [480], poor dental hygiene (for review see [481]) or other causes of chronic infections [482, 483]. Since depression and AD share not only a disturbed AHN but also neuroinflammation as a potential causative feature, Berk et al. asked a question also relevant for the UTAD, “So depression is an inflammatory disease, but where does the inflammation come from?” Their answer: Modifiable risk factors [484], which, in essence, can also be argued for AD.

As further depicted in Fig. 2, HPA-activation and therefore cortisol hypersecretion, a biomarker of MCI and early AD [178], leads to Aβ overproduction. Insufficient sleep as well as a lack of physical or social activity inhibit Aβ clearance via the BBB [259, 485]. Both, increased production as well as decreased clearance lead to an accumulation and formation of Aβ to neurotoxic oligomers. Furthermore, extracellular matrix (ECM) ecto-protein kinases and phosphatases [486] were found to enhance the aggregation and toxicity of the Aβ-species [487]. How this emerging field connects to current behavioural deficiencies or environmental toxins is not yet clear but it would be surprising if the physiology of the ECM activities would not be as highly regulated as all the other processes.

As indicated by the circular arrow in Fig. 2, toxic Aβ promotes its own generation by at least two mechanism. 1) Aβ as well as tau protein, the capital agents for the senile plaques and intracellular neurofibrillary tangles, are also called ‘prionoids’ indicating that proteins exhibit prion-like properties [488], which is characterized by propagation of protein misfolding [489], hence they share key biophysical and biochemical characteristics with prion diseases. 2) Oligomeric Aβ enhances pro-amylogenic cleavage of APP through the activation of GSK-3β, which also causes detrimental phosphorylation of tau [126]. All these pro-neurodegenerative processes interact and promote hippocampal shrinkage as well as cortical thinning. Additional vicious circles develop in the early phases of AD-pathogenesis, making it often difficult to distinguish cause and effect. Nevertheless, once vicious circles are initiated, they tend to accelerate the detrimental process on many different levels. Besides the viscous circle of depression (continuous black arrows), Fig. 2 contains several others (dashed black arrows):

- Sleep disturbances, as discussed above, increase toxic Aβ accumulation by different mechanisms. But toxic Aβ also impairs sleep [490], which at least in the early phase of AD might be a significant cause of memory deficits.
- Both arteriosclerosis and AD might be linked to similar behavioural deficiencies, which might explain the high degree of comorbidity [491]. In addition, arteriosclerotic hypoperfusion leads to an activation of pro-amylogenic APP-processing and reduced Aβ clearance, hence an increase in Aβ build up in the brain [492], whereas Aβ tends to accumulate in the blood vessel walls, leading to oxidative stress, inflammation and endothelial dysfunction, thereby enhancing the arteriosclerotic process [493].
- Toxic Aβ disturbs the function of the BBB, making a breakdown of this critical barrier both a consequence as well as an intermediate link in the causal chain of the pathogenic process [462], for instance by allowing bacterial or viral infections to enter the brain and accelerate disease progression [494, 495].
- Both toxic Aβ and chronically elevated cortisol concentrations enhance hippocampal (temporal lobe) insulin resistance [496]. Neuronal insulin resistance aggravates the impairment of AHN, which compromises via the “vicious cycle of depression” the patients’ ability to lifestyle alterations, which are required to recover neuronal insulin sensitivity, like engaging in physical and social activity or taking up a nutrient-rich diet.
- Aβ-activated GSK-3β not only leads to more Aβ but also phosphorylates tau, which also was shown to behave like a prion [497], thereby driving neurodegeneration. Neuronal damage and prionic aggregates aggravate neuroinflammation, which in turn enhances neurodegeneration [498]. Hyperphosphorylated tau also causes oxidative stress, which in turn leads to tau-hyperphosphorylation [129].

It is obvious, that any successful AD therapy needs to interrupt all significant self-maintaining and disease-accelerating vicious circles. How this can be accomplished will be outlined in a therapeutic proposal below. Such a scheme must include the removal of any type of environmental toxin that might inhibit AHN and entertain and aggravate the disease process:

- Tobacco smoke diminishes AHN and promotes gliogenesis in rats [499]. Hence, heavy smokers (2 packs a day) have a 2.6 fold increased AD risk [500]. But also electronic cigarettes, even if they would only contain nicotine as an ingredient (which they do not), are unhealthy and most likely AD-promoting, as nicotine impairs neurogenesis and plasticity of hippocampal neurons [501].
- Alcohol intake of two drinks or more per day accelerates AD by about 2 to 3 years and, when combined with heavy smoking (20 cigarettes per day) by about 4 to 6 years. And for those, carrying an ApoE4 allele, which aggravates the consequences of unhealthy lifestyle choices (as detailed above), the onset of AD is on average 10 years earlier [502]. High alcohol intake was shown to efficiently block AHN in an non-human primate model, with the effect lasted for 2 months even after alcohol discontinuation [503]. The lasting alcohol-induced reduction in AHN paralleled an increase in neural degeneration by nonapoptotic mechanisms. Interestingly, abstaining from alcohol was also seen as a risk factor in the past [504], but this finding has not been confirmed [505]. Hence, drinking small amounts of alcohol might not be statistically harmful, but most likely does not decrease the risk of developing AD.
- Trans fatty acids (TFAs), either from processed food (fried products and many fast-food sources) or from whole-fat dairy and ruminant meat products are implicated in AD [506]. Both sources were shown to equally efficiently increase low density lipoprotein (LDL)-cholesterol [507] and cause negative health effects [508]. TFAs drive the mortality risk from cardiovascular diseases [509] and increase the rates of cognitive decline in the elderly, again, irrespective of the source [510, 511]. Epidemiological studies starting with healthy participants showed that TFAs raise insulin resistance, blood pressure and cause also chronic inflammation [508], all well accepted causal risk factors for AD. Furthermore, TFAs inhibit the conversion of n-3 PUFAs into DHA, limiting their accumulation in the brain [512]. DHA depletion has been shown to result in decreased BDNF levels [513], in impeded productive AHN [514] and in neuroinflammation [308]. A recent in-vitro study provided convincing evidence that TFAs increase amyloidogenic processing of the amyloid precursor protein (APP), resulting in an overproduction of Aβ [515]. Moreover, TFAs were shown to enhance the oligomerization and aggregation of Aβ. Taken together, high intake of TFAs, independent of the source, might increase the AD risk by many avenues, also causing an earlier onset of the disease.
- Nitrosamines cause deficits in motor function and spatial learning, as well as neurodegeneration characterized by lipid peroxidation, increased levels of Aβ and p-tau, neuroinflammation and neuronal insulin resistance [516]. Nitrosamines are found in tobacco smoke. Significant levels of nitrosamines are also produced from nitrites and secondary amines found in many products, like processed meat and cheese preserved with nitrite pickling salt. This is another reason to avoid (mass produced) meat or cheese products.
- Bisphenol A (BPA) leads to unwanted hormonal activity and was shown to impair AHN, spatial learning and memory [517]. BPA is found in a variety of common consumer goods like water bottles. Epoxy resins containing BPA are used to line water pipes and for coatings on the inside of many food and beverage cans. Exposure to other bisphenols (B-Z) that are used as replacements (in order to advertise BPA-free products) show similar detrimental effects [518]. Hence BPA-free products are not necessarily safer and support the removal of all bisphenols from consumer merchandise [519].
- Pesticides: Roundup (a glyphosate-based herbicide) was recently shown to increase the lipid-peroxidation, glutamate excitotoxicity and oxidative damage in the hippocampus [520]. The widely used pyrethroid pesticide deltamethrin induces apoptotic signalling in the hippocampus and impairs AHN [521]. Similar effects were observed for Carbofuran, a carbamate pesticide [522]. These neurotoxic effect of widely used chemicals in conventional agriculture argues for the use of organic produced products for the prevention and the treatment of AD [4].
- Aluminium might act as an accelerator of the progression of [523]. Aluminium enhances pro-apoptotic signalling, reduces BDNF in the hippocampus and negatively affects spatial learning in animal models. In fact, every biochemical function in brain cells appears to be affected by aluminium (reviewed in [2]). In addition, aluminium may play crucial roles as a cross-linker in Aβ oligomerization [524]. Taken together, aluminium exposure should be avoided in prevention and treatment of AD.
- Methylmercury (MeHg) competes with selenium for its enzymatic binding sites and acts as a highly specific and irreversible inhibitor of selenoenzymes, which are required to prevent and even reverse oxidative damage throughout the body, in particular the nervous system. Inhibition of selenoenzymes appears to be the proximal cause of the pathological effects known to accompany MeHg toxicity [525]. Dietary selenium intake is inversely related to vulnerability to methylmercury (MeHg) toxicity, which explains why maternal ingestion of foods that contain MeHg in molar excess of Se has adverse child outcomes, whereas eating MeHg-containing but selenium-rich ocean fish results in improved child IQs [526]. Similarly, MeHg intake impairs hippocampal development and AHN [527] and is associated with delay in cognitive development [528] and AD [529]. Nevertheless, a recent study showed seafood consumption was associated with less AD neuropathology despite the increased mercury levels [58]. This controversial result might be explained by the fact that both DHA and selenium are contained in high concentration in seafood. Interestingly, in this study, ApoE4 carriers profited most of high seafood intake, or conversely, ApoE4 carriers are more harmed by a deficit in DHA and/or selenium consumption. Taken together, a diet low in MeHg is advised, but at least as important is a diet that keeps selenium and n-3 PUFAs (DHA and EPA) levels sufficiently high.
- Iron is essential for many metabolic processes, but, when left unregulated, is implicated as a potent catalyst of reactive oxygen species generation. Iron complexes with ferritin, the major cellular storage of this transition metal [530]. As outlined above, increased ferritin levels in the cerebrospinal fluid (CSF) are negatively associated with cognitive performance and predicted speed of MCI conversion to AD. Since ApoE4 (in contrast to ApoE2 and ApoE3) enhances iron uptake into the brain [59], carriers who consume large amounts of iron-rich animal products are particularly susceptible to elevated brain iron, enhanced ROS production and AD progression. This might be one of the reasons, why a diet low in animal products reduces AD risk; and pesco-vegetarians have the lowest mortality when compared to other common diets [531]. Individuals with MCI and high CSF-ferritin levels might delay conversion to AD by as much as 3 years by taking a chelating drug like deferiprone [532], according to the authors of the above mentioned CSF-ferritin-study. An alternative option with less side effects is treatment with α-lipoic acid (ALA) [533], which was shown to be highly effective in reversing oxidative stress arising from iron overload [534]. Due to the multitude of additional useful properties of ALA for the treatment of AD, ALA will be part of a therapeutic scheme suggested below.
- Copper like iron is an essential element for human growth and development. And likewise, excessive intake which leads to free copper contributes to neurotoxicity and impaired spatial memory by specific changes in the expression of synaptic proteins and hippocampal signalling pathways, which causes oxidative stress and neuronal apoptosis [535]. Inorganic copper from drinking water can be directly absorbed and elevate the serum free copper pool, thereby attributing to AD progression [536]. In case of high free copper levels, three regimens are advised: (1) Avoiding water contaminated with copper and supplements, (2) restoring normal zinc levels, as it was shown that elevating zinc levels significantly reduced serum free copper in AD patients [3] and (3) treatment with free copper-lowering ALA [537], as will be discussed in more detail below.
- Medications: Use of gastric acid inhibitor was significantly associated with the presence of vitamin B12 deficiency [538], a risk factor for AD. Meanwhile, it was found that these drugs increase the levels of Aβ in the brains of mice and are association with an increased risk of dementia in humans [539]. Increases in incident dementia are associated with long-term use (over three years) of anticholinergic drugs like for example the tricyclic antidepressant doxepin, the antihistamines chlorpheniramine and diphenhydramine, and bladder control drugs like for instance oxybutynin [540]. A case–control study showed an association between the use of benzodiazepines (used for sleep and anxiety control) and the risk of AD [541]. A recent study provided evidence that memory and hippocampal architecture of WeDi-treated rats was particularly vulnerable to short-term treatment with the benzodiazepine midazolam [542]. Again, this short number of examples must suffice to show that there is always the chance that artificial chemicals like many commonly used drugs might interfere with the complex pathomechanisms outlined by the UTAD, thereby increasing AD risk.

### AD prevention strategy

**Fig. 3:**
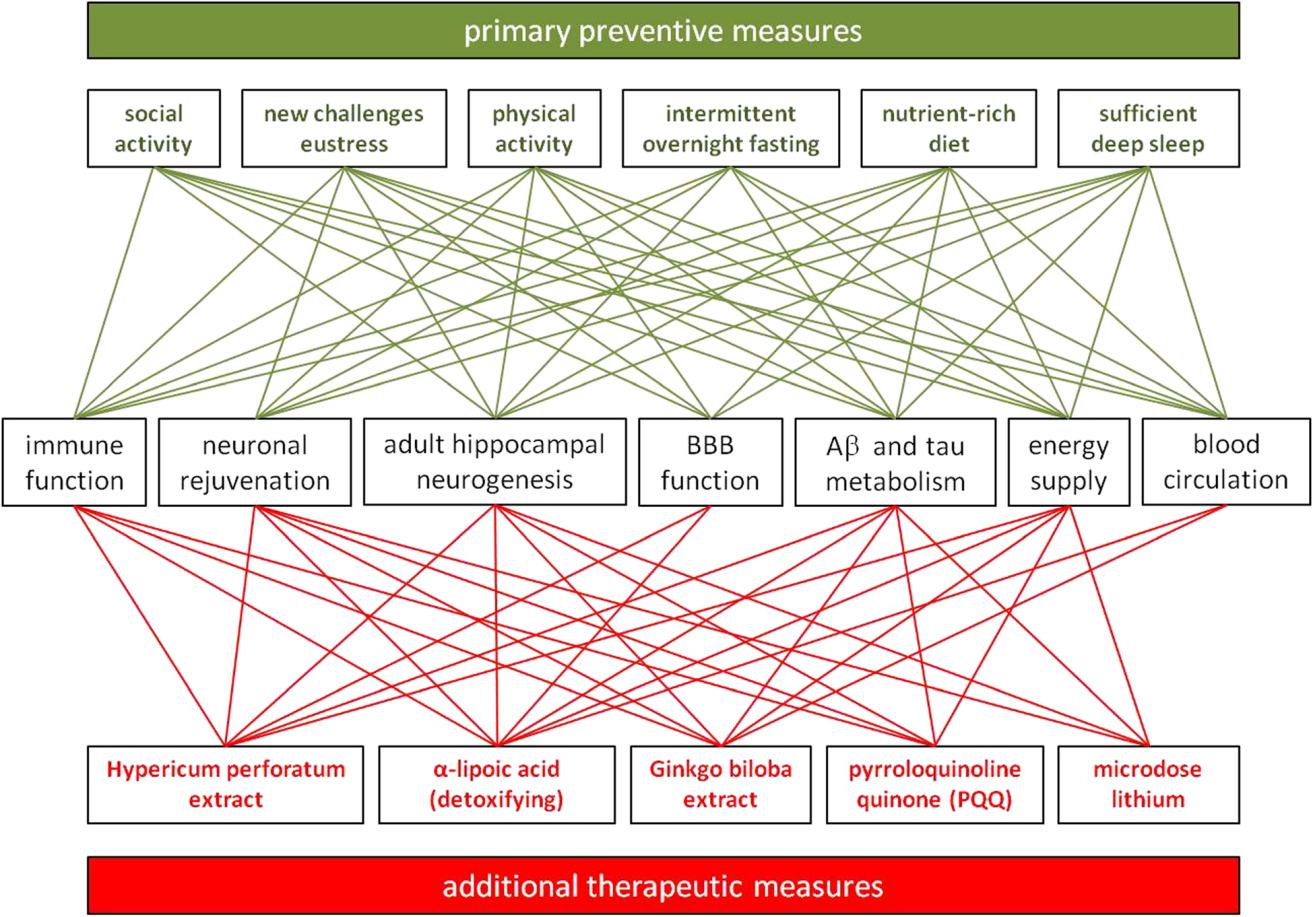
“A systems biological program for prevention and therapy of AD”. In order to support our genetic programs’ “interest” in maintaining lifelong mental health, the primary measures to prevent AD consist of a rich social life, daily new challenges which provide eustress and memorable experiences, physical activity and sufficient deep sleep, and adequate amounts of essential nutrients. As indicated by the green lines (and outlined in the main text), each of these measures have positive impacts, directly or indirectly, on key physiological systems or processes required for the preservation of mental health. In the case of an AD-diagnosis, these systems are not only compromised but, as outlined in Fig. 2 and detailed in the main text, lead to runaway phenomena. Hence, in addition to the implementation of the preventive measures and the correction of lifestyle-caused deficiencies, the proposed therapeutic interventions consist of a systemic combination of active components that were shown to interrupt the various vicious cycles that drive and accelerate the AD process. Each one was selected by its ability to interfere with a particular key pathophysiological process. But as indicated by the red lines and as outlined in detail in the main text, all of the suggested active components help to reactivate a variety of functions that are disturbed by the AD process

The UTAD provided a framework for the understanding that cognitive decline and Alzheimer’s (like any other culture-born) disease depend largely on modifiable risk factors. The top part of Fig. 3 schematically depicts the key areas where behavioural deficits can lead an increased AD risk. As indicated by the green lines, different behaviours impact AHN and other key physiological functions that are required for long-term brain health. But as predicted by the LOM, not a single one can completely replace or compensate for another that is lacking (although there might be some functional overlap between physical and intense social, i.e. oxytocin-inducing activities in respect to stimulating AHN). The essentiality of each factor in biological processes explains, why intervention trials that focus only on one aspect like physical activity [263] or solely on supplementation with n-3 PUFAs [543] lower AD risk only minimally or not at all.

One of the most comprehensive experiment in changing the lifestyle of an elderly population is the Finnish Geriatric Intervention Study to Prevent Cognitive Impairment (FINGER) [17]. The participants of the intervention group received dietary guidance and cognitive training and engaged in a social and physical activity program, whereas the control group received solely general health advise. According to the authors of the study, comprehensive outcome measurements suggested beneficial effects on both global cognition and cognitive domains highly relevant for everyday activities (e.g., executive functioning, processing speed, and complex memory tasks). Nevertheless, according to the proposed UTAD, even FINGER does not take into account a couple of important requirements for long-term brain health. According to the specifics of FINGER (http://www.alzheimersprevention.org/downloadables/FINGER-study-report-by-ARPF.pdf), it entailed for instance no specific program for stress-reduction or improving sleep quality, no instructions regarding the benefit of IMF or ketogenesis, and no monitoring (and therefore correction) of potential vitamin deficiencies (e.g. no homocysteine measurement). According to the recent study results, even the recommended daily dose of 400–800 units vitamin D is most likely is not sufficient to prevent AD [339]. In addition, no monitoring and correction of potential deficiency in essential trace elements was implemented.

But according to the UTAD, a program efficient in preventing cognitive decline needs to be individualized and comprehensive. It requires besides standard medical checkups a complete measurement of vitamins, essential trace elements as well as an analysis for the potential accumulation of environmental toxins outlined above. In case of high blood levels with toxic metals, ALA-treatment as an intervention-measure is advised. If, after a change in lifestyle, deficits remain (e.g., too low selenium concentrations in food produced in European countries caused by low amounts of selenium in the soil, or low vitamin D production in the skin when people live far in the north or in winter when simply not sufficient ultraviolet light reaches the skin [544], or insufficient lithium intake due to low concentration in tap water), specific dietary supplementation becomes an important part of a prevention strategy.

### Causal AD therapy

The pathogenesis of AD is progressive and therapeutic intervention should start as early as possible. AD advanced in stages from stage 1 or subjective cognitive decline (SCD) (for a detailed classification, see [545]), to stage 2 or MCI [546], to stage 3 or mild/early AD; stage 4 or middle/moderate AD, to stage 5 or late/severe AD.

A causal therapy according to the UTAD aims to eliminate all causal factors, identical to the AD prevention scheme outlined above. Due to the behavioural impairment of AHN, patients will present either with overt depression or at least exhibit cortisol hypersecretion. Therefore the cortisol status needs to be analyzed as well and regularly monitored during the therapy. Besides a guided return to a healthy lifestyle, the therapeutic regimen aims at interrupting the vicious cycles outlined above, which maintain and even accelerate the disease process. Once this is established, at least patients in stage 1 to stage 3 might fully recover episodic memory capability, as up to these early stages of AD, episodic memory deficits prevail and the patients do not yet suffer from AD-driven cognitive decline. This assumption is in line with the results from a recent study conducted by Dale Bredesen where 8 of the 10 patients that represented with stage 1 (3 patients), stage 2 (2 patients) and stage 3 (3 patients) recovered their episodic memory after adhering to a similar therapeutic scheme as it results from the UTAD (see below) [547]. The single stage 4 patient maintained his status and the one stage 5 patient was not treatable. As these results are rather case-based, they require confirmation by larger trials. But the success following the removal of individual deficits is fully in line with the UTAD proposed in this paper and its resulting implications for prevention and therapy. Similar improvements in cognition and episodic memory function in particular are meanwhile observed in the first three stage 3 AD-patients in a private practice (personal communication by W. Karner & B. Karner, Freiburg, Germany), which have started to implemented the treatment plan outlined herein.

I estimate that the therapeutic program takes about 6 months to complete. The calculation is based on several assumptions: First, it takes several weeks to implement the lifestyle changes, particularly if the unhealthy lifestyle is very comprehensive and deeply engrained. Second, AHN, once reactivated, requires several months to fully integrate new, adult-born DG-neurons. Furthermore, the timeframe is not much different from major depression treatment, where similarly an unproductive AHN needs to be targeted. In addition, in Bredesen’s pilot study, most patients were reported to show significant improvements and reversal of cognitive decline) after about 4–6 months. Similar observations are now being made in Freiburg, Germany (personal communication by W. Karner & B. Karner).

Besides reinitiating a productive AHN, the proposed therapy aims to reactivate the neuronal rejuvenation program and to interrupt the vicious cycles of AD that have been outlined above and are shown in Fig. 2. Furthermore, any lingering or overt infection should be treated, which includes dental care and treatment with probiotics to improve microbiome composition in order to reduce the acceleration of the AD process (see above). In addition to the individual preventive measures outlined above (upper part of Fig. 3), the following therapeutic measures are suggested (bottom part of Fig. 3):

### Hypericum perforatum extract (HPE)

As proposed by the UTAD, cortisol hypersecretion due to impaired AHN is a major link in the causal chain leading to AD. It not only drives the disease process, it also might reduce the patient’s compliance with the required therapeutic measures, in particular the openness and adherence to lifestyle changes. One way to overcome this depression-like predicament could be treatment with standard antidepressants like the SSRI-class, as they are also known to increase AHN as a key mechanism of action [548]. For example, one study showed that maturation and functional properties of young neurons are necessary for the anxiolytic/antidepressant activity of the chronically administered SSRI-type drug fluoxetine and thus explained the well-known delayed onset of therapeutic efficacy [549]. In another study, an SSRI-type antidepressant significantly reduced Aβ production in transgenic AD mice as well as in healthy humans [550]. In contrast to the slow process of hippocampal neurogenesis, the mechanism regulating Aβ production was found to be much faster. Apparently, stimulation of serotonin receptors quickly activates the extracellular-signal regulated kinase (ERK) signalling cascade, which was shown to be required for serotonin-dependent suppression of Aβ [551]. Activation of ERK signalling by an SSRI-type antidepressant increases non-amylogenic α-secretase activity [552] and reduces γ-secretase activity [553], both leading to lower Aβ levels.

An alternative to an SSRI-type antidepressant are extracts from Hypericum perforatum (HPE), which is also known as St. John’s wort. Based on a large meta-analysis of 29 clinical trials, a report of the Centre for Complementary Medicine Research in Munich, Germany, came to the following conclusion [554]: The available evidence suggests that HPE tested in the included trials a) are superior to placebo in patients with major depression; b) are similarly effective as standard antidepressants; c) and have fewer side effects than standard antidepressants. Meanwhile, the German S3 patient care guideline for unipolar depression published in 2015 regarded the evidence-based effect of 900 mg HPE as therapeutically equivalent to the lead SSRI Citalopram (20 mg/d) for the treatment of moderate major depression (http://www.leitlinien.de/mdb/downloads/nvl/depression/depression-2aufl-vers3-lang.pdf), whereby HPE is characterised by a superior tolerability. Similarly, another meta-analysis concluded that HPE does not differ from SSRIs by its efficacy but by its advantage in the management of major depressive disorder and by its higher patient compliance due to less adverse events [555].

HPE has many anti-AD-specific effects: (1) HPE efficiently reverses the anxiety/depressive-like state due to chronic corticosterone treatment [554]; (2) counteracts the reduced proliferation of progenitor cells in the murine hippocampus under chronic corticosterone treatment; (3) decreases the corticosterone-induced prevention in hippocampal stem cell proliferation in long-term; (4) and ameliorates corticosterone-reduced spine density (5). Tetrahydrohyperforin (THH), a synthetic derivative of hyperforin, one biologically potent constituent of HPE, (6) activates AHN both in wild-type mice and AD models [556]. (7) HPE treatment significantly decreases intracerebral Aβ42 levels (8), rescues neocortical neurons from toxic Aβ, (9) restores cognition to normal levels, and (10) and activates microglia in vitro and in vivo [557]. Mechanistically, the reduction of soluble Aβ42 species was shown to be a consequence of increased activity of the ABCC1-transporter, a member of the superfamily of ATP-binding cassette (ABC) transporters located in the BBB, which were found to play a fundamental role in Aβ excretion into the bloodstream. Likewise, HPE treatment reduced Aβ-accumulation in a double transgenic AD-model by activating ABCB1, another ABC transporter involved in Aβ export [558]. (11) In addition, THH was found to inhibit the amylogenic processing of APP [559]. By this mode of action, (12) THH prevented tau phosphorylation and synaptotoxicity in a murine AD model [560]. (13) And last but not least, mice treated with HPE showed increased levels of adiponectin in white adipose tissue and an insulin-sensitizing effect [561].

Taken together and as shown in Fig. 3 (red lines), HPE acts against multiple AD specific processes and vicious cycles. Nevertheless, although HPE is generally safe and in the USA even available without prescription, it should only be taken under the guidance of an experienced physician, as HPE is known for its significant induction of the detoxifying liver enzyme CYP 3A4 [562]. The therapeutic administration of a standard dose of about 1 g per day HPE may therefore result in diminished clinical effectiveness and increased dosage requirements for all CYP 3A4 substrates, which represent at least 50 % of all commercially available medications.

### α-lipoic acid (ALA)

ALA is an enzymatic cofactor, which is essential for aerobic mitochondrial energy production [563]. Though *de novo* synthesis in all animal cells appears to supply all the necessary ALA needed for its role in intermediary metabolism, ALA can also be efficiently absorbed from the diet. Only the (*R*)-(+)-enantiomer exists in nature and is superior in its biological activity [564]. The (*S*)-(−)-enantiomer as a by-product in chemical synthesis and many over-the-counter ALA-products might even block important biological activities of the natural form, like for instance ALA ability to reduce insulin resistance [565]. Hence when I further discuss ALA, I refer to the (*R*)-(+)-enantiomer.

Dietary ALA is safe in moderate doses and elicits a surprising array of metabolic and clinical effects that are of high interest in treating AD and related dementias (for review see [566]): (1) ALA and its reduced form dihydrolipoic acid (DHLA), which readily forms in the human organism, are highly efficient in chelating redox-active transition metals that might accelerate the AD process, as outlined above. Elimination of stockpiled toxins from the body by ALA may diminish their adverse impact on human biology and allow restoration of physiological function (for review see [567]). Importantly, neither ALA nor DHLA is capable of removing iron from aconites or copper from superoxide dismutase, implying that ALA supplementation modulates only the labile pool of redox active transition metals without causing metal depletion [568]. (2) Both ALA and DHLA are ideal antioxidants because they can easily quench radicals, scavenge ROS and lipid peroxidation products and inhibit the formation of hydrogen peroxide and hydroxyl radicals. For instance, ALA was shown to reduce glutamate- and oxidant-induced cell death by decreasing oxidative damage, increasing antioxidant defence and improving mitochondrial function [569]. (3) ALA regenerates other antioxidants, for example by increasing the level of reduced glutathione. (4) ALA also induces the enzymes of glutathione synthesis and other antioxidant protective enzymes. This mode of mitochondrial protection attenuates inflammation-induced impairment of AHN [570]. (5) ALA downregulates inflammatory processes and attenuates the release of free radicals and cytotoxic cytokines. As mentioned above, (6) ALA sensitizes the insulin receptor pathway [571], which leads to an increased glucose uptake in aged animals [572]. (7) ALA increases PGC-1α expression and activates a compromised cerebral blood flow, thereby protecting against focal ischemia [573]. Stimulating mitochondrial biogenesis is one of the important mechanisms for ALA to ameliorate cognitive dysfunction in aging and AD [574]. By activating PGC-1α, the effects of ALA are not organelle-limited, but reside on the functional and effective coordination of mitochondria-cytosol-nucleus communication that restores age-associated impairment of brain energy metabolism [572]. This might explain why ALA treatment in a stroke model was recently shown to be neuro-restorative and promoting functional recovery [575]. (8) ALA protects neurons from harmful effects of oligomeric Aβ, excess iron and other neurotoxins [576]. (9) ALA was also shown to stabilize the BBB [577]. Last but not least, (10) ALA increases acetylcholine (ACh) production, known to be reduced in AD brains [578].

This is of particular interest, as a recent study elucidated that endogenous ACh-signalling through α7-containing nicotinic receptors (α7-nAChRs) promotes maturation and integration of adult-born DG-neurons in the hippocampus. It indicates a profound role for the ACH/alpha7-nAChRs-system in AHN and conversely shows that α7-nAChR loss will cause progressive impairment in hippocampal circuitry and function over time as fewer neurons are added to the DG and those that are added integrate to a lesser extent [579]. In fact, increased release of ACh in the hippocampus due to physical activity might contribute to the stimulatory effect of exercise on AHN [580]. Hence physiological and/or pharmacological cholinergic stimulation(s) ameliorates cognitive decline even in aged animals, most likely by supporting AHN. By activation of choline acetyltransferase [581], ALA raises brain ACH-levels and thereby as well supports AHN. As summarized by Holmquist et al. [566]: ALA provides a prime rationale for symptomatic improvement of cholinergic cognitive dysfunction via stimulation of the ACh synthesis pathway.

One should be aware of the fact that ALA’s mode of action is much different from acetylcholinesterase inhibitors (AChEI) like donecepil [582], which raises ACH by inhibiting its degradation. This might explain ALA’s comparably much lower unwanted side effect profile. Due to the multiple additional functionalities (i.e., anti-inflammatory, antioxidant, carbonyl scavenging, metal-chelating, pro-energetic and neuroprotective properties) treatment with ALA promises to slow down the progression of AD. This is in contrast to AChEI in MCI, which, according to systematic review of randomised controlled trials was not associated with any delay in the onset of AD or dementia. Moreover, the safety profile showed, that the risks associated with AChEIs are not negligible [583]. Another, more recent meta-analyses conducted in 2015 provided evidence that treatment of MCI with AChEIs provides no benefit when compared with a control group. A small cognitive benefit was observed using behavioural therapies when compared with the control group, which might be used justify their current use. However, the authors conclude that the clinical significance of this small benefit remains uncertain and that current evidence does not support the use of AChEIs for treating MCI [584].

Treatment with 600 mg ALA daily improved MMSE scores in mild to moderate AD patients particularly if there was a comorbidity with type-2-diabetes [585], perhaps due to the additional capability of ALA of improving insulin sensitivity. In a study over 48 months, 600 mg ALA daily stabilized the cognitive functions in patients with mild dementia, with the disease progressing extremely slowly (MMSE: −0.6 points/year) [586]. Also in patients with moderate dementia, the rate of progression was dramatically lower than data reported for untreated patients or patients on AChEIs in the second year. Hence they conclude that despite the fact that their study was not double-blinded, placebo-controlled and randomized, treatment with ALA might be a promising “neuroprotective” therapy option for AD. Importantly, one has again to be aware that all these studies were carried out under the deficiency conditions, discussed above. Embedded in a therapeutic scheme that entails the preventive measures outlined in this review, the effect might be even better than merely stabilizing cognitive decline.

Although ALA is available as an over-the-counter nutritional supplement in many countries, most preparations contain the two possible enantiomers (*R*)-(+)-lipoic acid and (*S*)-(−)-lipoic acid as a racemic mixture. Patients should only be given (*R*)-(+)-lipoic acid under the supervision of an experienced physician, due to ALAs insulin-sensitizing ability. In this respect it is important to note that metal chelators like ALA might interfere with vitamin B12’s uptake mechanism from the GI tract [587]. Although this has not directly been shown for ALA, vitamin B12 should be monitored during long-term treatment since its deficiency might offset the benefits of ALA. Similarly, high doses of ALA might lead to a biotin deficiency, which requires for patients under ALA treatment to be supplemented with biotin [588]. Nevertheless, ALA in daily dosage of 600 mg is a safe multimodal drug for the treatment of AD particularly when applied under a comprehensive therapeutic plan.

### Extracts of *Ginkgo biloba* leaves (EGbs)

As outlined above, a disturbed microcirculation might be cause and effect of AD, forming a vicious circle between these two disease processes. Hence EGbs’ ability (1) to increase microcirculation and (2) to significantly improve the radical scavenging capacity even in elderly patients is of great therapeutic utility in AD [589]. These effects might be attributed to a multitude of potent pharmacologically active ingredients. For example, the standardized EGb-761 contains approximately 24 % flavonoid glycosides (for instance quercetin, kaempferol, isorhamnetin), 6 % terpenoids (of which 3.1 % are ginkgolides A, B, C, and J and 2.9 % is bilobalide), and 5–10 % organic acids (for review see [590]). Each of its constituents has different effects on the pathological mechanisms of AD (for review see [591]): In addition to the two effects mentioned above, (3) EGb-761 is able to reduce Aβ42-induced cell apoptosis [592], (4) Aβ-toxicity [593] and even (5) Aβ-production [594]; (6) EGbs positively modulate tau-metabolism [595]; (7) reduce oxidative stress and improve mitochondrial respiration. Particularly flavonoids, bilobalide and some of the ginkgolides (B and J) show a high neuroprotective capacity (for review see [596]). In diet-induced obese rats, (8) EGbs improve insulin signalling, dyslipidemia, and body adiposity, hence support dysfunctional energy metabolism [597]; (9) EGb-761 protected in ovariectomized rats as models of post-menopause against the decrease of cytochrome c oxidase activity, mitochondrial adenosine-5′-triphosphate and glutathione content in both platelets and the hippocampus, suggesting its potential role as a protective agent against central neurodegeneration in post-menopausal women [598]; (10) EGbs applied in a colitis-model dose-dependently inhibited the upregulation of TNF-α and IL-6, both inflammatory cytokines are involved in neuroinflammation [599]; (11) Last but not least, EGbs treatment showed a beneficial role on AHN even in aged mice with a significant increase in newborn DG-neurons with well-developed tertiary dendrites compared to controls [600].

Two recent meta-analyses concluded that treatment with EGb-761 at 240 mg/day over half a year stabilizes or slows decline in cognition, function, behaviour, and global change in cognitive impairment and dementia [601, 602]. Consequently, in January 2016, a group of AD experts presented the German S3 patient care guideline for dementia, where for the first time the use of EGBs (specifically EGb-761) was suggested for the treatment of patients with mild to moderate AD (http://www.dgn.org/images/red_leitlinien/LL_2016/PDFs_Download/038013_LL_Demenzen_2016.pdf). However, caution should be taken, when combining EGb with medicines metabolized by CYP3A4, as EGbs induce the expression of the gene [603].

Taken together, EGbs’ multiple modes of action relevant for AD, their potential to interrupt vicious cycles that are already active in the early phase of AD pathogenesis, and the fact, that EGbs like EGb-761 are generally very well tolerated at dosages of up to 240 mg/day even in aged patients taking multiple medications, renders them useful in a systemic therapeutic scheme as outlined in Fig. 3.

### Pyrroloquinoline quinone (PQQ)

PQQ was first discovered as a bacterial redox cofactor [604]. Since PQQ is also a potent plant growth factor, it is also part in the animal food chain. PQQ is reported to participate in a range of biological functions with apparent survival benefits (for example, improved neonatal growth and reproductive performance) [605]. There are also benefits from PQQ supplementation related to cognitive, immune, and antioxidant functions, as well as protection from cardiac and neurological ischemic events (for review see [606]). For instance, mice deprived of PQQ by a chemically defined diet show functional defects in connective tissue metabolism, are growth retarded and infertile [607], which suggests that PQQ plays a fundamental role as a growth factor. PQQ-deficient animals also show low cellular mitochondrial content, which is reversed when as little as 200–300 μg of PQQ/kg of diet are added [608]. Due to its nutritional importance, its inherent free-radical scavenging and redox modulator properties, and its coenzyme function in mammalian tissue [609], PQQ was proposed as a potential new B-vitamin [610]. Since PQQ showed neuroprotective effects in a cerebral ischemia model [611], it attracted pharmacological interest for the treatment of neurodegenerative diseases. Surprisingly, PQQ was found to inhibit the neurotoxic oligomerization of α-Synuclein, a causative link in the chain of events leading to Parkinson’s disease [612]. The anti-fibril-forming function of PQQ was soon extended to mouse prion protein and Aβ-42 [613]. Moreover, PQQ was shown to protect neuronal cells against Aβ-induced neurotoxicity [614].

Since diseases like AD are associated with mitochondrial dysfunction, PQQ supplementation may be of additional benefit since it efficiently stimulates mitochondrogenesis via activation of both, cAMP response element-binding protein (CREB) and PGC-1α [615]. As part of the proposed therapeutic scheme for AD, PQQ-supplementation would support the above outlined AD-preventing measures, namely IMF and physical activity, in activating mitochondrial biogenesis and NRJ. Indeed, dietary PQQ was shown to alter indicators of inflammation and mitochondrial-related metabolism in human subjects [616]. Furthermore, in a small clinical trial, 20 mg of PQQ daily for 8 weeks was shown to improve significantly measures of vigour, fatigue, tension-anxiety, depression, anger-hostility and confusion, as well as those for quality of life, appetite, sleep, obsession and pain [617]. In a double-blind, placebo-controlled comparative study, the mental status of middle-aged and elderly persons consuming foods containing PQQ for 12 weeks enhanced some of high-level cerebral functions including attention and discriminating and processing abilities [618]. Particularly in combination with microdosed lithium, PQQ might exert useful therapeutic effects in AD (see below).

### Lithium

As schematically shown in Fig. 3 and discussed above, daily intake of low amounts of lithium is an important preventive measure for maintaining brain health. Due to the interference of lithium in several self-maintaining signalling pathways in AD, daily intake of microdosed lithium (at least 300 μg with lithium-containing drinking water) is also of high therapeutic relevance: Lithium reduces toxic Aβ and p-tau generation, positively impacts mitochondrial rejuvenation and thereby energy metabolism. In addition, it appears to be important for productive AHN. Interestingly and in line with the therapeutic proposal in this work, Lazzara and Kim recently commented on the multimodal activity and therapeutic potential of lithium in neurodegenerative diseases [369]. They pointed out that high-dose lithium-only treatment may not be a suitable therapeutic option for neurodegenerative diseases due to inconsistent efficacy and potential side-effects. But low-dose lithium in combination with other potential or existing therapeutic compounds may be a promising approach to reduce symptoms and disease progression in neurodegenerative diseases.

Indeed, evidence for a powerful synergistic effect of low-dose lithium and PQQ supplementation was recently shown in a murine double transgenic AD model. The results showed that the combination at relative low dose exhibited more powerful effects in restoring impaired learning and memory, facilitating hippocampal LTP, and reducing cerebral Aβ deposition and p-tau level when compared to lithium alone. According to the authors, their study demonstrated the efficacy of a novel AD therapeutic strategy in targeting multiple disease-causing mechanisms through the synergistic effects of microdose lithium and PQQ [619].

Similarly, according to the UTAD the daily intake of sufficient amounts of essential trace elements like selenium and zinc, essential n-3 PUFAs DHA and EPA, vitamins etc. as part of a healthy diet is part of the therapeutic scheme. Nevertheless, in the initial phase of the therapy, all potential deficits need to be evaluated and treated immediately by supplementation until a change in diet eliminates the occurrence of those deficits.

## Summary and discussion

Over 50 thousand AD researchers worldwide continue to build an ever-growing amount of scientific data, each one being another piece of an unsolved puzzle. It appears that analytically dividing the puzzle in evermore pieces does not bring us closer to a complete picture. In order to find a path through the wealth of data and contradicting hypotheses, I made a basic assumption: I took for granted that the evolutionary theory is correct and therefore should serve as a firm foundation as well as a guide to reconcile conflicting interpretations. And indeed, the interpretation of AD as a modern epidemic started to make sense from this perspective and led to the UTAD, which delivers a logical explanation for the behavioural causes of AD, all known risk factors as well as an understanding of how AD might be overcome. Nevertheless, the search term “Alzheimer” retrieved far more than 80 thousand publications at the National Centre for Biotechnology Information (NCBI). It was therefore impossible to check each one for consistency with the herewith proposed UTAD as a behavioural deficiency disease. It was not even possible to discuss each one that I have studied. But whenever I found discrepancies, they were either explained by the design of the study or the (mis) interpretation of the results. Very often, these were a consequence of the false assumption that correlation equals causality, or normality equals naturalness (for instance, that aging of animals kept under unnatural conditions reflects natural aging or a natural course of AD; the same goes for human intervention or epidemiological studies).

The UTAD explains the evolutionary logic that led to human’s exceptional longevity and why our genetic program should protect us from ailments like AD or other mind-incapacitating diseases. This program like any other works best under the conditions to which it was tailored. Since modernization changed our way of living faster than our genetic program could adapt to it, and because productive AHN is particularly vulnerable to many culture-born deficiencies, the LOM as an important part of the UTAD easily explains why AD can be caused by a multitude of different individual deficiencies. Furthermore, the UTAD helps to understand the current epidemic of AD (as well as of major depression) in modern societies and the steady rise of its incidence and prevalence rates in emerging economies, which parallels the economic growth rate. But it also explains, why intervention trials in AD often fail or show only limited success. Instead of correcting all individual disease-causing deficits in their intervention group, they offer one for all solutions with a limited set of corrective measures. It is not surprising that usually no or only marginal success can be reported [16], since only those few individuals in an intervention group will benefit from the correction of a deficit who suffer from this (and only this!) particular deficit. If we want to win the fight against dementia, only an aggressive correction of all (!) individual risk factors (deficiencies) might, according to the UTAD, turn out to be a successful strategy.

In 2006, Brinton and Wang discussed that the causes of age-associated decline in AHN remain to be fully determined, but that loss in growth factors like FGF-2, IGF-1 and VEGF, in the microenvironment of the subgranular zone (SGZ) are prime contributors to the reduced neurogenic potential hypothesis [620]. What they were not aware of is that the neurogenic potential even at higher age is still there, following the results from a landmark study published in 2013 [150]. Proof of the persistence of the neurogenic potential up to higher age is even provided by analysis of post-mortem brains in late-stage AD [621]. Interestingly, some argue that this observed upregulation in AD-brains is evidence against the assumption that disease-related loss of AHN might be a key causal contributor to cognitive impairment in AD [622]. Hence, reactivation of AHN by removing AHN-impairing behavioural deficits, as proposed in this review, might not be a therapeutic option. But is this a valid objection? There is general consensus in the field that the generation of fully mature and functional neurons is impaired in AD [623], although both increases, decreases, no changes, and changes dependent on the stage of the disease have been described in AD patients and animal models (for review see [624]). Indeed, Aβ signalling through p-tau drives ectopic neuronal cell cycle re-entry in AD, causing neurodegeneration of the hippocampus by aberrant and non-productive neurogenesis [625]. As was shown, the resulting increase in proliferation of neural progenitor cells (NPCs) does neither increase the number of migratory neuroblasts nor that of differentiated neurons [626]. All these pathogenic effects are most likely attributed to the AHN-inhibiting neuroinflammatory microenvironment prevailing in AD, as well as an unchanged lifestyle that continues to deprive the hippocampus from behavioural stimuli and essential nutrients required for a productive AHN. For instance, the unproductive proliferation of NPC in AD might also be driven by the combined action of immunomodulators particularly acting in the late stage of the disease, like for instance transforming growth factor β1 (TGFβ1).

TGFβ1-signalling under physiological conditions protects neurons from potential Aβ-toxicity [627] and enhances AHN [628], primarily by upregulating the proliferation of NPCs and promoting the survival of newly generated neurons by supporting their functional differentiation, while, at the same time, maintaining the neurogenic stem cells (NSC) in a quiescent stage [629]. In contrast, under pathological conditions like chronic neuroinflammation (caused by the multiple behavioural deficiencies, as has been discussed above), chronically elevated levels of TGFβ1 contribute to disease progression [630, 631]. For example, chronic exposure to TGFβ lengthens the G1 phase of the cell cycle in activated stem cells, thereby impairing cell cycle progression of neural progenitors [632]. At the same time, high cortisol and toxic levels of Aβ and many other pathogenetic effects due to inflammation harm the maturation and integration of the adult-born neurons in the DG-network.

The assumption that AHN could be of great therapeutic potential is not new and raised the hope that transplanting NSC might one day cure AD [68, 633]. The optimism generated a flurry of studies in which the effects of stem cell transplantation on cognitive decline in animal models of AD were investigated [634]. Some experiments provided the unexpected evidence that proliferating stem cells injected in the murine hippocampus even ameliorated Aβ-induced neurotoxicity and cognitive decline by inhibiting apoptotic cell death and oxidative stress [635]. While this was certainly encouraging news, one fundamental question remains: Why would we want to inject exogenous NSC in the hippocampus, if patients, at least in the early stages of AD, could benefit from reactivated “endogenous” AHN in the subventricular zone of the hippocampus? Of course, since AHN is restricted to the formation of mostly granule cells and some interneurons in the DG [205], activation of productive AHN by lifestyle intervention in already heavily compromised late stage of AD-patients is less likely to be disease modifying (large parts of the brain being irreversibly affected) and practically difficult (compliance compromised). This is the main reason why I am arguing for early diagnosis.

Meanwhile, more and more experts in the field demand pleiotropic interventions to prevent or even cure AD [636, 637]. They realize that monotherapy targeting early single steps in the complicated cascade with agents that solely inhibit production, clearance, or aggregation of Aβ is not sufficient. But without a UTAD, as I propose it in this review, their arguments are less convincing to a research community, where the majority is still driven by the hope of finding the “magic bullet”, a therapy that does not require any changes in lifestyle. In particular, since most proposals for intervention lack a comprehensive explanation for all the complex processes that run more or less simultaneously in AD, the question which molecular targets and/or behavioural deficits need to be addressed and what therapeutic interventions are required has so far been answered rather arbitrarily. But this is detrimental, as every researcher usually favours his or her own concept of AD, which leads to many conflicting hypotheses. So, the public receives a steady flow of information about the pros and cons of such partial explanations, which (in line with the LOM) might never be the basis for fully preventing or curing AD.

Fighting AD does not mean fighting human nature. Claiming that AD is mainly and fatally caused by aging per se unjustifiably frightens the public. Furthermore, who would be willing to change his way of living if researchers belittle the effects. Therefore we should rather encourage a change to a healthy lifestyle and offer early diagnostic services in order to correct AD-causing deficiencies as early as possible.

## Conclusion

The proposed Unified Theory of Alzheimer’s Disease (UTAD) defines AD as a behavioural deficiency disease, emanating from a discrepancy between the natural requirements, which are explained by long-lasting humans natural life history, and our modern lifestyle, which is explained by humans’ very recent cultural (technological, social and economic) history. These deficiencies interfere with neuronal rejuvenation and adult hippocampal neurogenesis, both being required to maintain mental health, as well as with many other physiological processes and systems, which aggravate and accelerate mental decline. The UTAD provides an encompassing explanation of how the multiple well-known risk factors (including environmental and genetic) interact to cause AD and clarifies their impact on the aetiology and pathogenesis of AD. Furthermore, the UTAD makes clear why prevention strategies based on the correction of only one or a few risk factors fail or show only limited success, and similarly, why treatment strategies, which ignore the primary pathological causes and their correction might continue to be ineffective. Conversely, the UTAD argues for a systemic biological approach based on the law of the minimum governing essential processes required for lifelong mental health in order to prevent sporadic AD with high probability. In addition, the logical framework provided by the UTAD allows the development of a curative regimen, which is based on the individualized correction of detrimental deficits and a multi-pronged, systems-biological approach with the aim to interrupt key pathological processes in AD. Hence this review based on my theoretical findings argues that current prevention strategies need to be reconsidered and fundamentally changed. Furthermore, monotherapeutic treatment strategies that ignore the primary causes and deficiencies should be replaced by a systems-biological approach that is to be initiated as early as possible in the course of disease aiming at eliminating all disease-causing factors in order to give the patient a fighting chance to reverse cognitive decline.

## Abbreviations

AA, arachidonic acid; Aβ, amyloid beta; AcAc, acetoacetate; ACh, acetylcholine; AChEI, acetylcholinesterase inhibitor; ACTH, adrenocorticotropic hormone; AD, Alzheimer’s Disease; AGEs, advanced glycation end products; AHN, adult hippocampal neurogenesis; ALA, α-lipoic acid; alpha7-nAChRs, alpha7-containing nicotinic receptors; ALS, amyotrophic lateral sclerosis; BBB, blood brain barrier; BDNF, brain-derived neurotrophic factor; βOHB, D-β-hydroxybutyrate; CA, cornu ammonis; CSF, cerebrospinal fluid; CCR, chronic caloric restriction; CR, corticosteroid receptor; CRF, corticotropin releasing factor; DG, dentate gyrus; DHA, docosahexaenoic acid; DHLA, dihydrolipoic acid; DPA, docosapentaenoic acid; EE, enriched environment; EGbs, extracts from Ginkgo biloba leaves; EPA, eicosapentaenoic acid; EPO, erythropoietin; ERK, extracellular-signal regulated kinase; FDG-PET/CT, 2-deoxy-2-[fluorine-18] fluoro- D-glucose integrated with computed tomography; FGF-2, fibroblast growth factor 2; GDNF, glial cell-derived neurotrophic factor; GH, growth hormone; GMH, grandmother hypothesis; GSK-3α, glycogen synthase kinase-3α; GSK-3β, glycogen synthase kinase-3β; HC, hippocampus complex; HCAR2, hydroxycarboxylic acid receptor 2; HDAC, histone deacetylases; HMIT, hippocampal memory index theory; HPA axis, hypothalamic–pituitary–adrenal axis; HPE, Hypericum perforatum extract; IGF-1, insulin-like growth factor 1; Il-1, interleukin-1; IL-6, interleukin-6; IMF, intermittent fasting; LDL, low density lipoprotein; LEC, lateral entorhinal cortex; LTP, long-term potentiation; LOM, law of the minimum; LRP1, low density lipoprotein receptor-related protein 1; MCTGs, medium-chain triglycerides; MEC, medial entorhinal cortex; MeDi, Mediterranean diet; MeHg, methylmercury; MCI, mild cognitive impairment; MMSE, mini–mental state examination; MRI, magnetic resonance imaging; mTOR, mammalian target of rapamycin; NFL, national football league; NGF, nerve growth factor; NPD1, neuroprotectin D1; NPY, neuropeptide Y; n-3 PUFA, omega-3 polyunsaturated fatty acid; n-6 PUFA, omega-6 polyunsaturated fatty acid; NRJ, neuronal rejuvenation; PGC-1α, peroxisome proliferator-activated receptor γ coactivator 1α; PRH, perirhinal cortex; PUFAs, polyunsaturated fatty acids; REM, rapid eye movement; ROS, reactive oxygen species; SCD, subjective cognitive decline; SSRI, selective serotonin reuptake inhibitor; SWS, slow-wave sleep; TFAs, trans fatty acids; THH, Tetrahydrohyperforin; TNF-α, tumour necrosis factor-alpha; UTAD, unified theory of AD; VEGF, vascular endothelial growth factor; WeDi, Western diet
